# Catechol Derivative‐Based Bioadhesives: Molecular Design for Precision Medical Adhesion

**DOI:** 10.1002/advs.202521272

**Published:** 2026-01-07

**Authors:** Xueyu Wang, Zixin Yang, Shan Wang, Weiwei Tan, Zhirui He, Qinyu Duan, Huanan Wang, Tao Chen, Shanshan Hu

**Affiliations:** ^1^ Chongqing Key Laboratory of Oral Diseases Chongqing Municipal Key Laboratory of Oral Biomedical Engineering of Higher Education Chongqing Municipal Health Commission Key Laboratory of Oral Biomedical Engineering The Affiliated Stomatological Hospital of Chongqing Medical University Chongqing 401147 P. R. China; ^2^ Key State Laboratory of Fine Chemicals School of Bioengineering Dalian University of Technology Dalian 116024 P. R. China

**Keywords:** adhesion, bioinspired adhesives, catechol chemistry, cohesion, tissue regeneration

## Abstract

Acute tissue injuries demand bioadhesives with strong wet adhesion, biocompatibility, and mechanical adaptability. While mussel‐inspired catechol‐based adhesives hold promise, current designs often neglect the role of molecular diversity in tuning cohesion–adhesion dynamics. Inspired by the hierarchical branching of trees, where branch length and position determine fruit composition, we developed a structurally tunable bioadhesive platform by grafting five DOPA‐derived catechol derivatives—varying in side‐chain length and substituents—onto polyvinyl alcohol (PVA) via esterification. Computational, spectroscopic, structural, and mechanical analyses revealed that side‐chain length and substituents critically modulate the adhesive and cohesive properties of the hydrogels. Among these, the PVA‐CA system exhibited superior cohesion and adhesion across diverse substrates, attributed to the extended side chain and conjugated double bond of caffeic acid (CA) that enhance intramolecular packing and interfacial interactions. Ex vivo adhesion on porcine/canine cardiac, pulmonary, and intestinal tissues, along with in vivo studies in hepatic defect and skin wound models, confirmed strong adhesion, biocompatibility, and improved tissue regeneration of PVA‐CA hydrogel. This work establishes a programmable molecular design strategy for next‐generation wet‐tissue adhesives with broad biomedical potential.

## Graphical abstract

This study utilizes tree‐inspired side‐chain engineering to graft five catechol derivatives onto PVA, systematically revealing how side‐chain length and substituents regulate adhesion and cohesion properties. Among them, the PVA‐CA patch demonstrates superior tissue adhesion, biocompatibility, and promotes regeneration, establishing a programmable molecular design strategy for next‐generation wet‐tissue adhesives with broad biomedical applications.

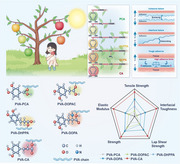

## 1 Introduction

Acute organ and tissue injuries, such as extensive trauma, uncontrolled hemorrhage, and complex fractures are responsible for millions of deaths annually worldwide [1, 2]. Conventional surgical closures, including suturing and stapling, remain standard practice but require prolonged operative time and experienced personnel [3]. Moreover, these techniques are frequently associated with postoperative complications such as leakage and inflammation [3]. Bioadhesive materials have thus emerged as promising alternatives, offering simplified application, reduced tissue trauma, and a lower risk of complications [4, 5]. However, current bioadhesives face critical limitations that hinder their clinical translation. In particular, their adhesive strength is substantially compromised under dynamic and wet physiological conditions (e.g., blood and interstitial fluids), leading to dilution or poor retention [2, 6]. Furthermore, a fundamental trade‐off exists between biocompatibility and adhesive efficacy: synthetic crosslinkers (e.g., cyanoacrylates, glutaraldehyde) exhibit strong adhesion but cause cytotoxicity, while natural alternatives (e.g., proteins, carbohydrates) are biocompatible but exhibit inferior adhesive performance [7, 8]. Compounding these issues, the intrinsic interfacial heterogeneity of various tissues (e.g., mucosa, bone, myocardium) necessitates tunable adhesive properties tailored to organ‐specific biomechanical and healing requirements [9]. These challenges underscore the urgent need for bioadhesives with wet‐environment functionality, mechanical adaptability, and bioresponsive behavior.

Nature offers compelling paradigms for overcoming these limitations [10]. Marine mussels, for instance, achieve strong adhesion under extreme wet and saline conditions through the secretion of mussel foot proteins (Mfps) enriched in 3,4‐dihydroxyphenylalanine (DOPA) [11, 12, 13]. The adhesive mechanism is primarily governed by catechol groups in DOPA, which facilitate both covalent bonding (e.g., Michael addition, Schiff base formation) and non‐covalent interactions (e.g., metal coordination, hydrogen bonding, π–π stacking), culminating in hierarchically reinforced networks through synergistic cohesion–adhesion effects [14, 15, 16]. Inspired by this, DOPA‐based biomimetic hydrogels have been developed by grafting catechol groups onto polymeric backbones such as polyvinyl alcohol (PVA), hyaluronic acid (HA), and polyethylene glycol (PEG) [17, 18, 19]. For instance, our precious study demonstrated enhanced transmucosal drug delivery via a PVA‐DOPA adhesive for buccal tissues [17]; Xue et al. reported a dual‐crosslinked dopamine‐isothiocyanate‐modified HA adhesive promoting peripheral nerve regeneration [18]; and Fujita et al. developed an oxime‐crosslinked PEG hydrogel with catechol moieties for improved cardiac retention and pericardial adhesion prevention [19].

**SCHEME 1:**
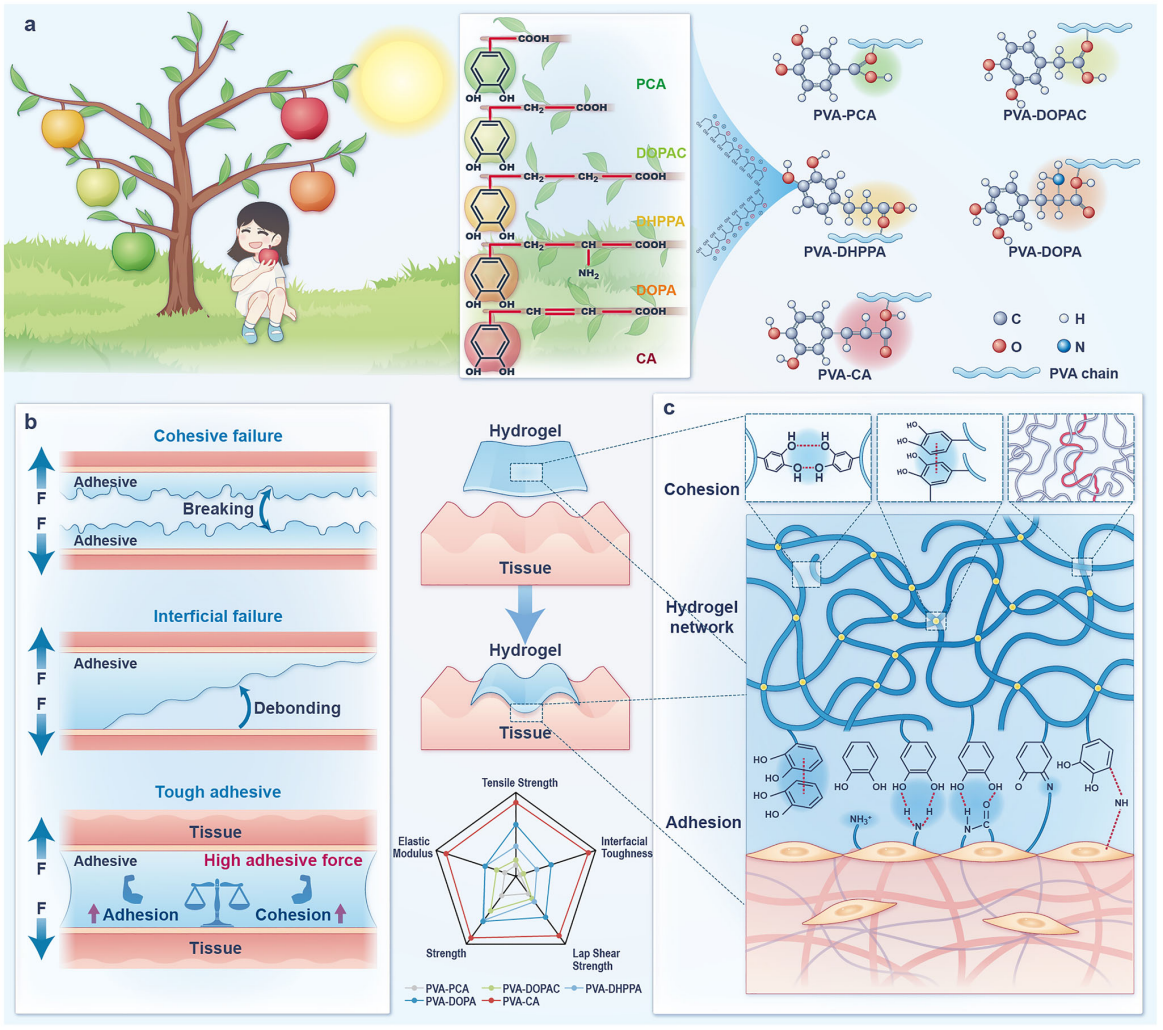
Schematic illustration of the inspiration, structural formula, cohesion/adhesion mechanisms, and applications of hydrogel adhesive. (a) Schematic diagram of inspiration and structural formulas of five catechol derivatives and adhesive systems. (b) Schematic illustration of different adhesion mechanisms of hydrogel adhesives under external force, and (c) strategies for enhancing cohesion and interfacial adhesion through various physico‐chemical methods.

Despite these advances, most mussel‐inspired adhesives focus on a singular molecule design, largely neglecting the broader influence of structural diversity among catechol derivatives [20]. Yet, the superior underwater adhesion of mussels derives not solely from isolated chemical functionalities but from synergistic and spatially organized interactions [21]. Recent studies have begun to explore this structural dimension. Cheng et al. demonstrated that the number and position of phenolic groups critically influence adhesion strength [22], whereas Shen et al. elucidated the effect of DOPA chirality on material properties [23]. However, these investigations still remain confined to DOPA or catechol groups, failing to explore the broader potential offered by diverse molecular architectures. In addition, although it is well recognized that adhesive strength arises from both cohesive and adhesive forces, these two mechanisms are often considered as an inseparable whole, obscuring their distinct contributions [20, 24]. To address these two longstanding challenges in biomimetic adhesive design, we draw inspiration from arboreal systems, where branch architecture governs the distribution of sunlight and nutrients, leading to fruit with varied sugar content (Scheme 1). Analogously, we hypothesize that modulating substituent topology and side‐chain length in catechol derivatives may enable control over interfacial microenvironments, thereby tuning both cohesion and adhesion in a programmable manner.

Here, we systematically evaluated five catechol derivatives with varying side‐chain lengths or substituents: 3,4‐dihydroxybenzoic acid (PCA), 3,4‐dihydroxyphenylacetic acid (DOPAC), 3‐(3,4‐dihydroxyphenyl) propanoic acid (DHPPA), DOPA, and 3‐(3,4‐dihydroxyphenyl) prop‐2‐enoic acid (caffeic acid, CA). These compounds were grafted onto PVA backbones via esterification to generate a panel of biomimetic adhesives. Density functional theory (DFT) calculations were conducted to predict how substituent‐induced structural variations influence intramolecular interactions and interfacial binding capabilities. The chemical structures of the synthesized polymers were confirmed using Fourier transform infrared (FT‐IR) spectroscopy, UV‐visible (UV–Vis) spectroscopy, and proton nuclear magnetic resonance (^1^H NMR) spectroscopy. Multiscale mechanical and interfacial characterizations—including atomic force microscopy (AFM), wide‐angle X‐ray scattering (WAXS), and small‐angle X‐ray scattering (SAXS)—enabled quantification of internal network cohesion and dynamic tissue adhesion performance. Among the five adhesive systems, the PVA‐CA hydrogel exhibited superior cohesive and adhesive properties. This enhancement is attributed to the extended side chain and conjugated double bonds of CA, which facilitate denser intramolecular packing and stronger interfacial hydrogen bonding with wet biological substrates. Ex vivo adhesion tests using porcine/canine cardiac, pulmonary, and intestinal tissues confirmed robust adhesive strength of PVA‐CA system. Furthermore, in vivo studies in porcine hepatic defect and linear skin wound models demonstrated excellent biocompatibility and enhanced tissue regeneration. By integrating bioinspired structural motifs, molecular‐level design, and structure‐function correlations, this work establishes a programmable materials platform for next‐generation wet tissue adhesives. It also offers new mechanistic insights into the interfacial regulation of catechol‐derived systems, paving the way for precision‐engineered adhesives tailored to clinical needs.

## 2 Results and Discussion

### 2.1 Design and Structural Analysis of Various Catechol Adhesive Systems

**FIGURE 1:**
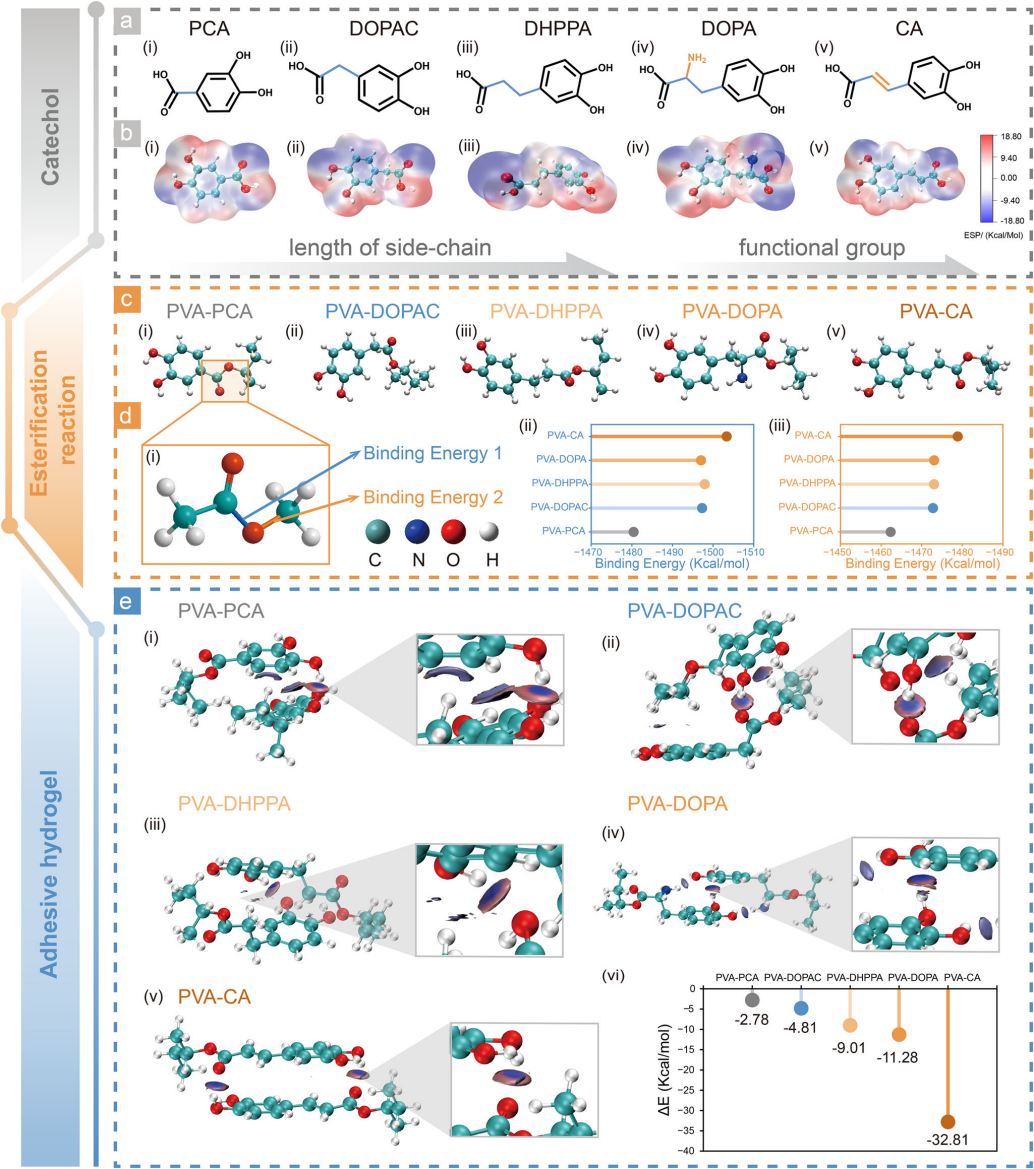
Design and structural analysis of catechol‐based bioadhesives using DFT. Chemical structures (a) and ESP (b) distributions on the molecular surfaces of five catechol derivatives, selected based on variations in side‐chain length and functional groups. (c) Fully optimized geometries and binding energies (d) at two ester bond sites for products derived from the esterification of the five catechol derivatives with PVA. (e) IGMH analysis of the interchain interactions within the five adhesive systems.

DOPA, the most extensively investigated catechol derivative, possesses hydroxyl groups at the 3‐ and 4‐ positions that facilitate strong adhesive interactions, alongside carboxyl and amino groups that support diverse polymerization pathways [25, 26]. Based on the structural features of DOPA, namely the adjacent 3,4‐dihydroxy moieties and the carboxyl group at the 1‐position, we selected five catechol derivatives with varying side‐chain lengths (PCA, DOPAC, DHPPA) and different substituent groups (DOPA and CA) (Figure 1a).

To explore their adhesive potential, we firstl analyzed the electrostatic potential (ESP) distributions on the molecular surfaces of these derivatives. ESP maps highlight electrophilic and nucleophilic sites by identifying regions of most positive and negative potential, respectively. For all derivatives, the hydroxyl hydrogen atoms exhibited positive ESP, while the oxygen atoms displayed negative ESP, suggesting strong potential for hydrogen bonding and thereby favorable adhesive performance [27] (Figure 1b). Based on our previous studies, we employed esterification reactions to graft these catechols onto PVA, forming adhesive hydrogel networks [17] In this process, the carboxyl groups of catechols react with hydroxyl groups of PVA to form ester bonds that act as crosslinking points, with bond energy serving as a key determinant of polymer stability and cohesion. As presented in Figure 1c, the fully optimized geometries of the five esterified products, determined via DFT calculations at the B3LYP/6‐311G* level. Among these, the PVA‐CA conjugate exhibited the highest binding energies at two ester bond sites (−1503.331 and −1478.860 kJ/mol; Figure 1d), indicating superior chemical stability and mechanical robustness.

To further validate the predicted differences in polymerization behavior, we analyzed interchain interactions using the Independent Gradient Model based on Hirshfeld partition (IGMH). The δ ginter isosurfaces (Figure 1e) and corresponding scatter plots (Figure S1) revealed the presence of noncovalent interactions in all five adhesive systems. Specifically, blue isosurfaces indicate attractive forces such as hydrogen bonding between catechol hydroxyls, interchain hydrogen bonding of the hydrogel backbone and π–π stacking between aromatic rings, both of which are essential to interchain cohesion [28,29]. In addition, the gray areas in the corresponding scatter plots are difficult to find, indicating that the Van der Waals forces between the hydrogel chains are very weak. Additionally, DFT‐based interaction energy calculations supported these findings. The PVA‐CA system exhibited a significantly higher interaction energy (−32.81 kJ/mol) compared to PVA‐DHBA (−2.78 kJ/mol), PVA‐DOPAC (−4.81 kJ/mol), PVA‐DHPPA (−9.01 kJ/mol), and PVA‐DOPA (−11.28 kJ/mol) (Figure 1e (vi)), indicating a markedly stronger network‐forming capacity and mechanical integrity. Collectively, these data highlight that structural features of catechol derivatives, particularly CA, critically govern the cohesive and adhesive performance of resulting polymers.

### 2.2 Synthesis and Characterization of Various Catechol Adhesive Systems

**FIGURE 2:**
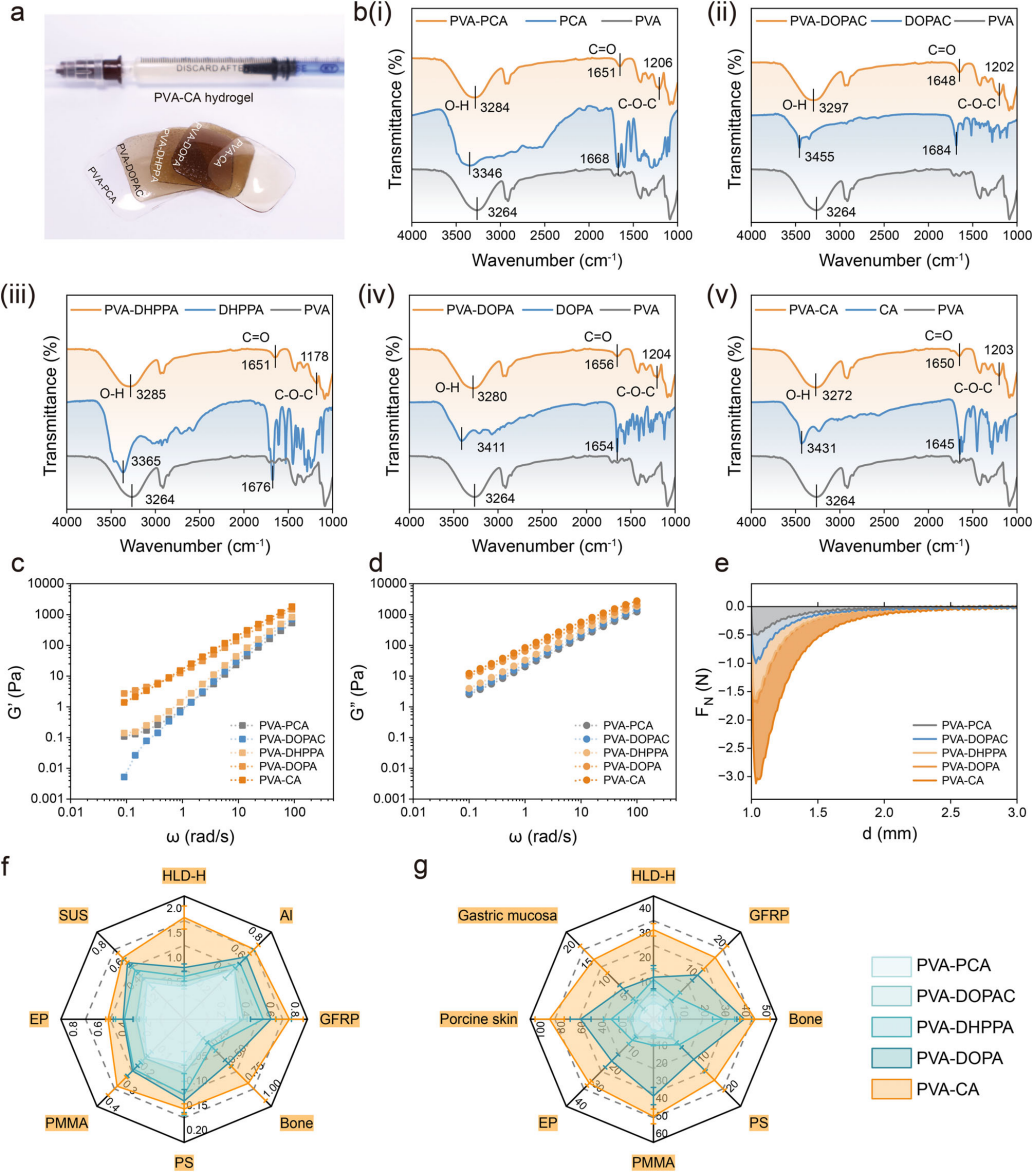
Characterization and adhesive strength of the five adhesive systems. (a) Photographs of films formed by the five adhesive systems. (b) FTIR spectra of the five adhesive systems. (c) Storage modulus (G′) and (d) loss modulus (G″) of the five adhesive systems. (e) Vertical normal adhesion force of the five adhesive systems. (f) Lap shear adhesive strength, (g) tensile adhesive strength of the five adhesive systems, measured on various substrates (n = 5).

Firstl, PCA, DOPAC, DHPPA, DOPA, and CA were grafted onto PVA via esterification to synthesize the PVA‐PCA, PVA‐DOPAC, PVA‐DHPPA, PVA‐DOPA, and PVA‐CA polymers. As shown in Figure 2a, these polymers could be processed into either gels or films. Scanning electron microscopy (SEM) analysis revealed 3D porous structures in all hydrogels (Figure S2a‐e). The synthesis of the five adhesive systems was confirmed through FT‐IR spectroscopy, UV–Vis spectroscopy, and ^1^H‐NMR spectroscopy. In the FT‐IR spectra, after esterification, the carboxyl (‐COOH) stretching bands of the catechol monomers (initially at 1645–1684 cm^−1^) shifted to carboxylate (‐COO^−^) vibration peaks at 1648–1656 cm^−1^. Meanwhile, new ester C‐O‐C asymmetric stretching peaks appeared at 1178–1206 cm^−1^ [30]. These characteristic changes confirm the successful synthesis of the PVA‐PCA, PVA‐DOPAC, PVA‐DHPPA, PVA‐DOPA, and PVA‐CA polymers. Furthermore, a shift of hydroxy groups (─OH) from 3264 cm^−1^ for pure PVA toward higher wavenumbers, suggesting the formation of new cross‐linking network between the PVA and catechol. In the UV–Vis spectra (Figure S3a), π–π transitions, in accordance with molecular orbital theory, generated characteristic absorption peaks at 255/290 nm for benzoic acid derivatives due to UV absorption by π electrons in the outer orbitals of benzene ring. Similarly, DOPAC, DHPPA, and DOPA displayed catechol absorption at 280 nm, while CA exhibited peaks at 290 and 320 nm, confirming the covalent conjugation of catechol monomers to the PVA chains [31, 32]. Additionally, ^1^H NMR spectra (Figure S3b) displayed the characteristic peaks of catechol groups at δ 6.3–7.6 ppm, further supporting the successful synthesis of the catechol‐PVA hydrogel systems [33, 34]. The degree of substitution was calculated by calculating the phenyl signals of the catechol moieties with the methylene signals of the PVA backbone [17]. As shown in Table S1, the esterification degree of PVA was approximately 8.8%–9.3% and the grafting efficiency was 35.2%–37.2%, both showed no significant difference between PVA‐CA and the other four groups (n = 3 independent samples).

Rheological measurements were conducted to further assess the mechanical properties of the hydrogel systems. The storage modulus (G'), which is directly related to the crosslinking density and stiffness of the network, revealed that the PVA‐CA group exhibited high storage and loss moduli, as shown in the rheological data (Figure 2c,d). This indicates a high crosslinking density and significant viscous dissipation within the PVA‐CA network [35]. Additionally, the viscosity (Figure S3c) and normal adhesive force (Figure 2e) of the catecholic hydrogel systems were measured. The PVA‐CA hydrogel demonstrated superior viscosity and normal adhesive force compared to the other four groups, suggesting enhanced adhesive properties.

To further compare the adhesive strength of the five adhesive systems, firstl, five hydrogel patches were tested for swelling (Figure S4). The 7‐day swelling rate of PVA‐CA in PBS was 109.5 ± 5.7%, indicating that it can avoid irritation to adjacent fragile tissues and maintain lasting and strong biological adhesion [36]. Also, ex vivo experiments were performed across various bonding interfaces. Shear tests tensile tests and interfacial toughness test were conducted using a universal testing machine to assess the adhesive strength on multiple substrates, including stainless steel (SUS), aluminum (Al), fiberglass (GFRP), plastic (PS), acrylic (PMMA), resin (EP), synthetic stone (HLD‐H), bovine bone (Bone), procine skin and procine gastric mucosa [37]. The results showed that the PVA‐CA hydrogel exhibited the highest interfacial toughness, shear and tensile strength across all tested interfaces, outperforming the other four groups (Figure 2f,g and Figures S5–S7).

### 2.3 Cohesive Force and Structural Analysis of Various Adhesive Systems

**FIGURE 3:**
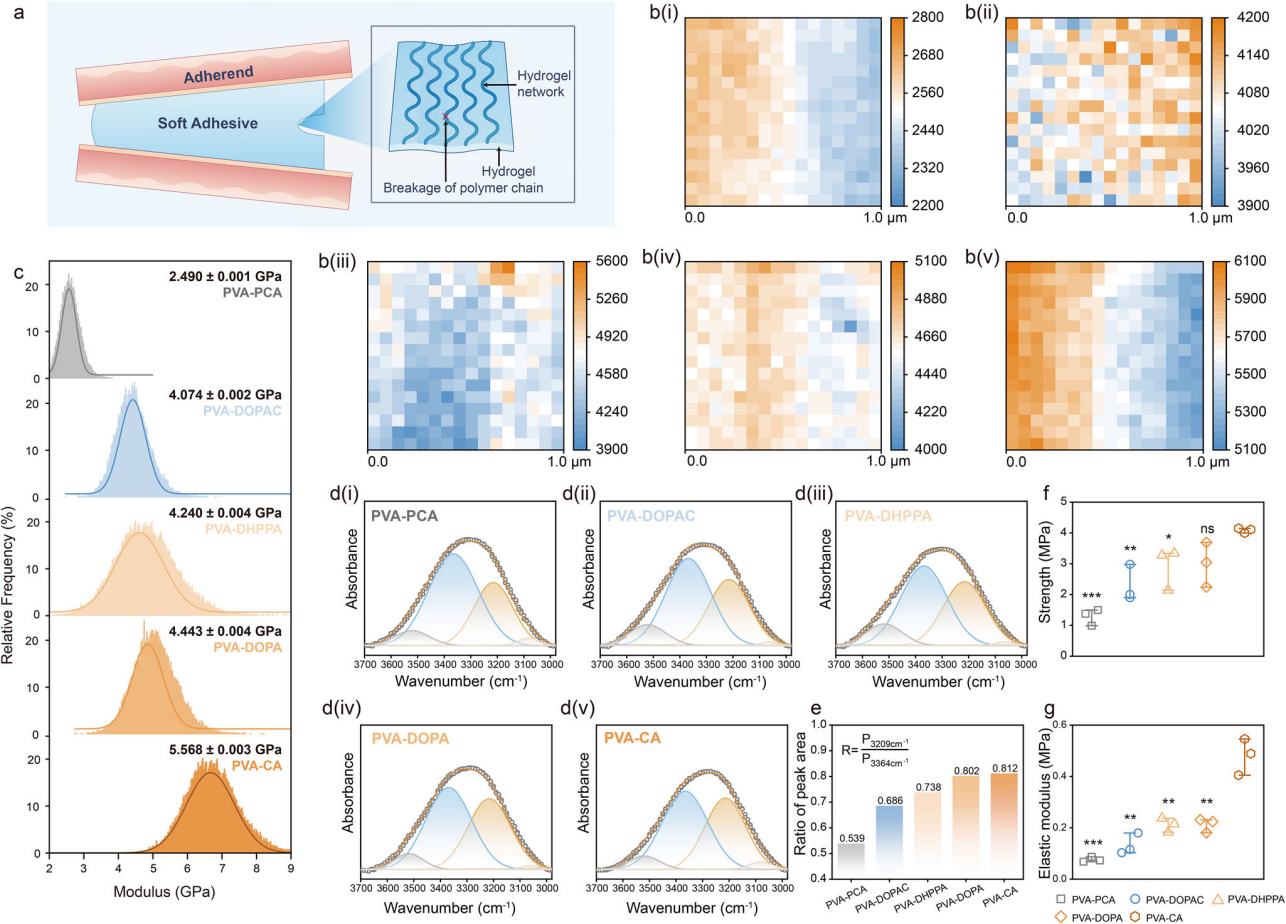
Cohesive force and mechanical properties of various catechol‐based bioadhesive. (a) Schematic diagram of hydrogel cohesion failure. (b) Surface modulus (MPa) distribution of the five adhesive systems. (i) PVA‐PCA, (ii) PVA‐DOPAC, (iii) PVA‐DHPPA, (iv) PVA‐DOPA, (v) PVA‐CA. (c) Gaussian simulation spectrum of Young's modulus (GPa). (d) Deconvoluted FTIR spectra of five adhesive systems in the wavenumber range of 3000 to 3700 cm^−1^, as well as the area ratio of the peaks located at 3209 and 3364 cm^−1^. (e) The two peaks are assigned to hydroxyl groups on catechol hydrogels that form hydrogen bonds with catechol hydrogels and water, respectively. Uniaxial tensile strength (f) and elastic modulus g) of hydrogels. (n = 3 per group, *p < 0.05, **p< 0.01, ***p < 0.001, ns, not significant, one‐way ANOVA, Tukey's post hoc analysis).

The adhesive strength of bioadhesives is widely recognized to be influenced by two distinct factors: cohesive force and adhesive force. Cohesive force refers to the internal force that maintains the integrity of the hydrogel network, while adhesive force describes the bonding strength between the hydrogel adhesive and the target tissue surfaces. Adhesive failure can result from either interfacial debonding (adhesive failure) or internal rupture due to poor mechanical stability (cohesive failure) (Figure 3a) [38, 39, 40]. To address these factors, this study systematically investigates the differences in adhesive strength among catechol‐based systems by evaluating both cohesive and adhesive contributions. We begin by examining how the structure of various bioadhesives influences their cohesive force, employing techniques such as AFM, FT‐IR, SAXS, and WAXS.

First, AFM was used to probe the micromechanical properties of the five adhesive systems [23]. The coefficient of variation (CV) of the AFM DMT (Derjaguin‐Muller‐Toporov) modulus indicates that the hydrogel membrane surface is uniformly distributed (Table S2).  As shown in Figure 3b and Figure S8, Young's modulus maps were acquired over 1 × 1 µm^2^ areas (n = 65,536 data points) on the hydrogel film surfaces, alongside corresponding topography maps generated by averaging Young's modulus values over adjacent 4 × 4 pixel regions. The Gaussian fit shows a single peak distribution, and the main peak is highly concentrated, indicating that the material is homogeneous [23]. The surface Young's modulus of the PVA‐CA film averaged 5.568 ± 0.003 GPa, significantly higher than that of the other four groups (Figure 3c). Based on IGMH analysis, this enhancement is likely associated with increased the enhanced interaction force within the PVA‐CA hydrogel network.

The hydrogen bonding content in the five adhesive systems was further quantified through FT‐IR peak deconvolution. FTIR fitting was performed in the O─H stretching region (3700 cm^−1^ to 3000 cm^−1^), where two characteristic peaks at 3209 cm^−1^ and 3364 cm^−1^ were attributed to interchain hydrogen bonding within catechol chains and chain‐water hydrogen bonding, respectively (Figure 3d). The area ratio of the peak at 3209 cm^−1^ to that at 3364 cm^−1^ was calculated to quantify the relative proportions of interchain hydrogen bonding versus chain‐water bonding [41, 42]. As shown in Figure 3e, the PVA‐CA group exhibited the highest proportion of interchain hydrogen bonding, followed by the PVA‐DOPA group, which corroborated the IGMH calculations. This suggests that the PVA‐CA hydrogel enhances its micromechanical cohesion through increased energy dissipation via sacrificial interchain hydrogen bonds. Furthermore, the five adhesive systems were molded into standardized dumbbell‐shaped specimens for uniaxial tensile testing. The resulting elastic modulus and tensile fracture strength are presented in Figure 3f,g, respectively. PVA‐CA demonstrated an elastic modulus of 0.48 ± 0.07 MPa and a tensile fracture strength of 4.09 ± 0.08 MPa, both significantly higher than those of the other four groups. The increased density of internal interchain hydrogen bonds within the PVA‐CA hydrogel network contributes to its enhanced cohesive strength and macroscopic toughness [39, 40].

**FIGURE 4:**
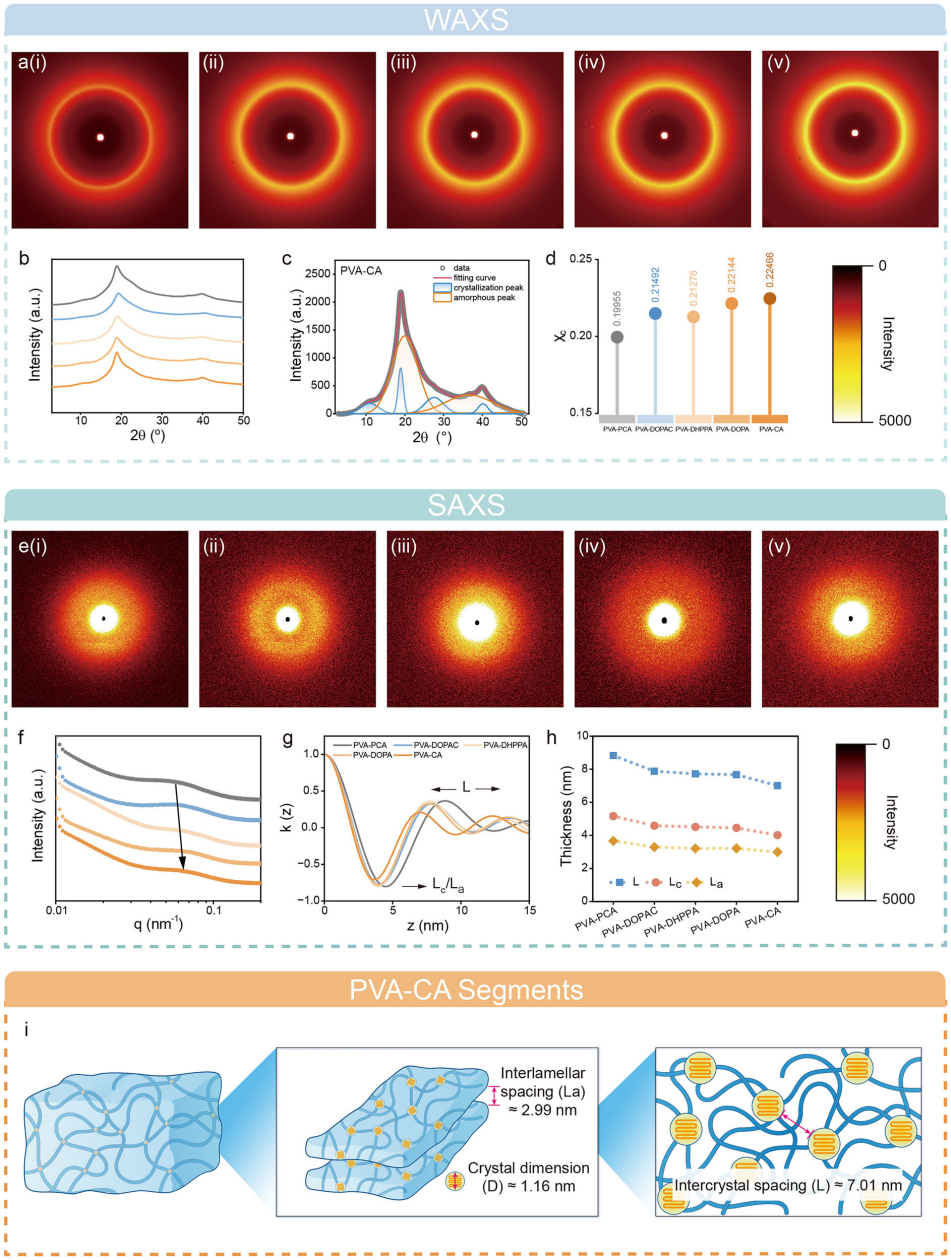
Structure analysis of different catechol‐based adhesives using WAXS and SAXS. (a) 2D WAXS patterns of the five adhesive hydrogels films. (i) PVA‐PCA, (ii) PVA‐DOPAC, (iii) PVA‐DHPPA, (iv) PVA‐DOPA, (v) PVA‐CA. (b) Corresponding 1D integrated WAXS curves. (c) Multipeak fitting of the 1D integrated WAXS curve of PVA‐CA. (d) The crystallinity (χ_c_) of five adhesive systems. (e) 2D SAXS patterns of the five adhesive hydrogels films. (f) Corresponding 1D integrated curves of the 2D SAXS patterns. (g) Typical curve of the correlation function, by which the lamella thickness dc can be obtained. (h) Change of L (interlamellar spacing), L_c_ (lamellar thickness), and L_a_ (interlamellar distance). (i) Schematic illustration of the crystal dimension (D), L, and La within the PVA‐CA hydrogel samples.

To further explore the structural characteristics across various scales within the adhesive systems, we employed WAXS and SAXS. These techniques reveal hierarchical structural features ranging from the nanometer to sub‐nanometer scale, providing insights into the differences in cohesive strength among different bioadhesives [43]. WAXS provides information on crystalline structural differences. Figure 4a displays the 2D WAXS patterns for the five adhesive hydrogels films. These patterns show isotropic peaks. After integration, the corresponding 1D integrated WAXS curves are presented in Figure 4b. Quantitative analysis of the 1D integrated WAXS curves was performed using multi‐peak curve fitting, as shown in Figures 4c and S9 the scattering angle range of 10° to 30° was selected, and lattice planes corresponding to different crystals were identified. Based on the deconvolution results, the crystallinity index (χ_c_) is shown in Figure 4d. The diffraction peak intensities of the catechol‐based adhesive systems exhibit slight variations, with the PVA‐CA hydrogel displaying the highest crystalline intensity. This suggests that enhanced intermolecular forces resulting from increased crystallinity in the PVA‐CA group facilitate the formation of a denser network structure, thereby improving cohesive strength. The dimensions of catechol‐based adhesive systems were calculated according to Figure 4b (Figure S10).

SAXS analysis further elucidated the variations in nanoscale network structures, contributing to the differences in cohesive strength among the catechol‐based adhesive systems. Figure 4e presents the isotropic 2D SAXS scattering rings of the five adhesive hydrogels films. The 1D integrated SAXS curves (scattering intensity I(q) vs. scattering vector q) were derived from the azimuthal integration of the 2D SAXS patterns as a function of q (Figure 4f). The scattering peak positions for the five adhesive systems progressively shifted toward higher q‐values (PVA‐PCA q ∼ 0.060 nm^−1^; PVA‐DOPAC q ∼ 0.062 nm^−1^; PVA‐DHPPA q ∼ 0.066 nm^−1^; PVA‐DOPA q ∼ 0.069 nm^−1^; PVA‐CA q ∼ 0.070 nm^−1^). According to the Bragg law approximation (d ≈ 2π/q), smaller characteristic dimensions (d) correspond to larger q‐values, which are observed on the right side of the curves, indicating reduced nanoscale structural features within the hydrogels [44]. Additionally, the long period (L) was calculated from the q_max_ of the 1D integrated curves using the Bragg equation. The lamellar thickness (L_c_) was determined through Lorentz correction, as shown in Figure 4g. The interlamellar distance (L_a_) was subsequently calculated by subtracting L_c_ from L [45]. The final results are summarized in Figure 4h. The long period L can be used to evaluate the nanoscale periodic structure within hydrogels, representing the average distance between adjacent repeating units. The results indicate that the PVA‐CA hydrogel film exhibited the smallest long period (L ≈ 7.01 nm), reflecting enhanced crystalline structure integrity and lamellar thickening. Correspondingly, both lamellar thickness (L_c_) and interlamellar distance (L_a_) reached their minimum values in PVA‐CA (L_c_ ≈ 4.02 nm; L_a_ ≈ 3.00 nm), which indicated that PVA‐CA improved the degree of crystallinity and strengthened the physical interactions between the lamellae (Figure 4i) [46, 47].

In summary, nano‐ and sub‐nanoscale analysis using SAXS and WAXS reveals that the crystallization reinforcement mechanism endows the PVA‐CA hydrogel with superior cohesive strength. This enhancement improves the macroscopic toughness of the gel, making the PVA‐CA hydrogel less prone to deformation or detachment from adhesive surfaces during adhesion. These properties better meet the practical application requirements for bioadhesives. However, excessive cohesive force may lead to interfacial adhesive failure [48, 49]. Therefore, our subsequent research will further explore the interfacial adhesive force in different adhesives.

### 2.4 Adhesive Force of Various Catechol‐Based Bioadhesives

**FIGURE 5:**
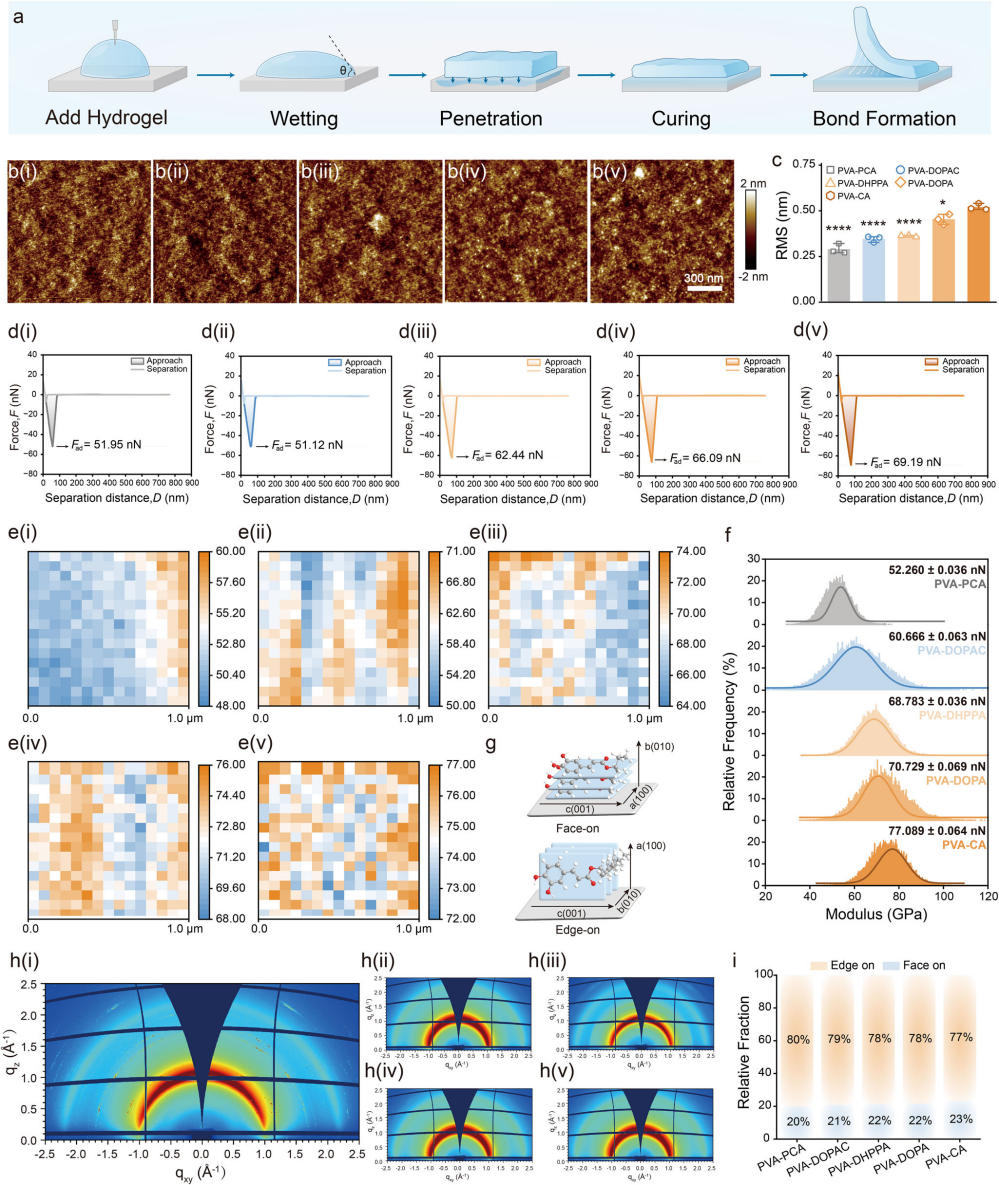
Adhsive force of different catechol‐based adhesives. (a) Schematic diagram of the bonding process. (b) AFM images of the surface modified by (i) PVA‐PCA, (ii) PVA‐DOPAC, (iii) PVA‐DHPPA, (iv) PVA‐DOPA, (v) PVA‐CA. (Scanning area size = 1.6 × 1.6 µm, scalebar = 300 nm). (c) Surface roughness of the five adhesive hydrogels films. ((n = 3 per group, *p < 0.05, **p< 0.01, ***p < 0.001, ****p < 0.0001, one‐way ANOVA, Tukey's post hoc analysis). (d) Measured normal interaction forces of AFM tips with the five adhesive systems. (e) Adhesion distribution of the five adhesive hydrogels films (nN). (f) Gaussian simulation spectrum of adhesion. (g) Schematic of the face‐on and edge‐on packing represented by PVA‐CA. (h) 2D‐GIWAXS patterns of (i) PVA‐PCA, (ii) PVA‐DOPAC, (iii) PVA‐DHPPA, (iv) PVA‐DOPA, (v) PVA‐CA. i) Relative crystal fractions with the edge‐on and face‐on orientation in the five adhesive systems.

The bonding process involves several sequential stages: wetting, adsorption, penetration, curing, and bond formation, with effective wetting serving as the fundamental prerequisite for robust adhesion (Figure 5a) [50, 51]. To compare the wetting capabilities of the five adhesive systems, we measured the water contact angles on various substrates (Figure S11). The results demonstrated that the PVA‐CA hydrogel exhibited lower contact angles across a range of substrates—including stainless steel, aluminum, glass fiber, plastic, acrylic, resin, artificial stone sheets, and bovine bone—indicating higher surface affinity and superior wetting performance [22]. Adhesive bonding occurs through conformal penetration into the microstructures of rough surfaces, establishing intermolecular interactions at the interface. To further investigate this, we compared XPS spectra of pristine catechol‐based adhesives with interfacial layers of adhesives bonded to a glass substrate (Figure S12a–e), where the substrate interface (glass) was pulverized and mixed with the hydrogel. The results revealed altered peak areas in the O 1s spectra for the hydrogel/glass blends, with C‐O/C = O area ratios of 5.88, 5.91, 7.08, 7.17, and 7.68 for the five adhesive systems, respectively, compared to 5.47, 5.48, 5.93, 6.10, and 6.22 for the pristine hydrogels. This systematic increase in ratio confirms hydrogen bond formation during interfacial interactions, with PVA‐CA showing the most significant hydrogen bonding contribution (Figure S12f) [52].

During the curing process, the hydrogel undergoes physical changes or chemical reactions, establishing cohesive strength and bonding the adherends together. The cohesive force of the different hydrogels has been demonstrated in previous experiments (Figures 2 and 3). Upon curing, the final bond forms with sufficient mechanical strength. AFM analysis revealed uniformly distributed protrusions on the mica surface after bond formation, displaying a granular surface morphology that confirmed the successful loading of all catechol‐based adhesive hydrogels onto the substrate surface (Figure 5b and Figure S13). The results showed that the PVA‐CA hydrogel exhibited the highest root mean square (RMS) roughness (0.541 nm) among the groups (Figure 5c). This increased roughness indicates the formation of effective mechanical interlocking structures during curing, enhancing the adhesive strength [23].

To further compare interfacial adhesive forces, force curve measurements were performed using AFM [53]. The five adhesive systems were deposited onto mica substrates for AFM force experiments, as illustrated in Figure 5d. In these typical force spectroscopy experiments, the silicon tip approached the surface at a constant speed and retracted after reaching a constant peak force. As expected, the PVA‐CA group showed the highest force. Figure 5e,f display the interactions between the AFM tip and the adhesives, demonstrating that the adhesive force of PVA‐CA on the AFM tip (77.089 ± 0.064 nN) exceeded that of PVA‐DOPA, PVA‐DHPPA, PVA‐DOPAC, and PVA‐PCA (70.729 ± 0.069 nN; 68.783 ± 0.036 nN; 60.666 ± 0.063 nN; 52.260 ± 0.036 nN, respectively).

Additionally, to examine the alignment of the cured catechol‐based adhesive systems relative to the interface, we conducted 2D grazing‐incidence wide‐angle X‐ray scattering (GIWAXS) experiments on dried hydrogel systems on silicon substrates. The 2D GIWAXS patterns of the catechol‐functionalized hydrogel films (Figure 5h) display diffraction rings corresponding to the out‐of‐plane (q_z_) and in‐plane (q_xy_) directions. To quantitatively analyze the differences in molecular orientation within the films, integration was performed over a polar angle (χ) range of 0° to 90°(Figure S14). The diffraction intensities for χ = 0°‐30° and χ = 30°‐90° were attributed to the face‐on and edge‐on orientations, respectively (Figures 5g–i). The lamellar stacking structures of the adhesive hydrogels films primarily exhibited an edge‐on orientation, but the ratio of edge‐on to face‐on orientation varied among the groups. The PVA‐CA film showed a relatively higher face‐on ratio of 23%, indicating a larger proportion of molecules with their conjugated planes parallel to the substrate (i.e., the π‐π stacking direction perpendicular to the substrate) compared to the other groups [54, 55]. As a result, the PVA‐CA hydrogel likely achieves enhanced interfacial molecular ordering, improving interfacial interactions and leading to increased adhesion strength.

### 2.5 Interactions and Adhesion Performance of Catechol‐Based Bioadhesives to Tissues

**FIGURE 6:**
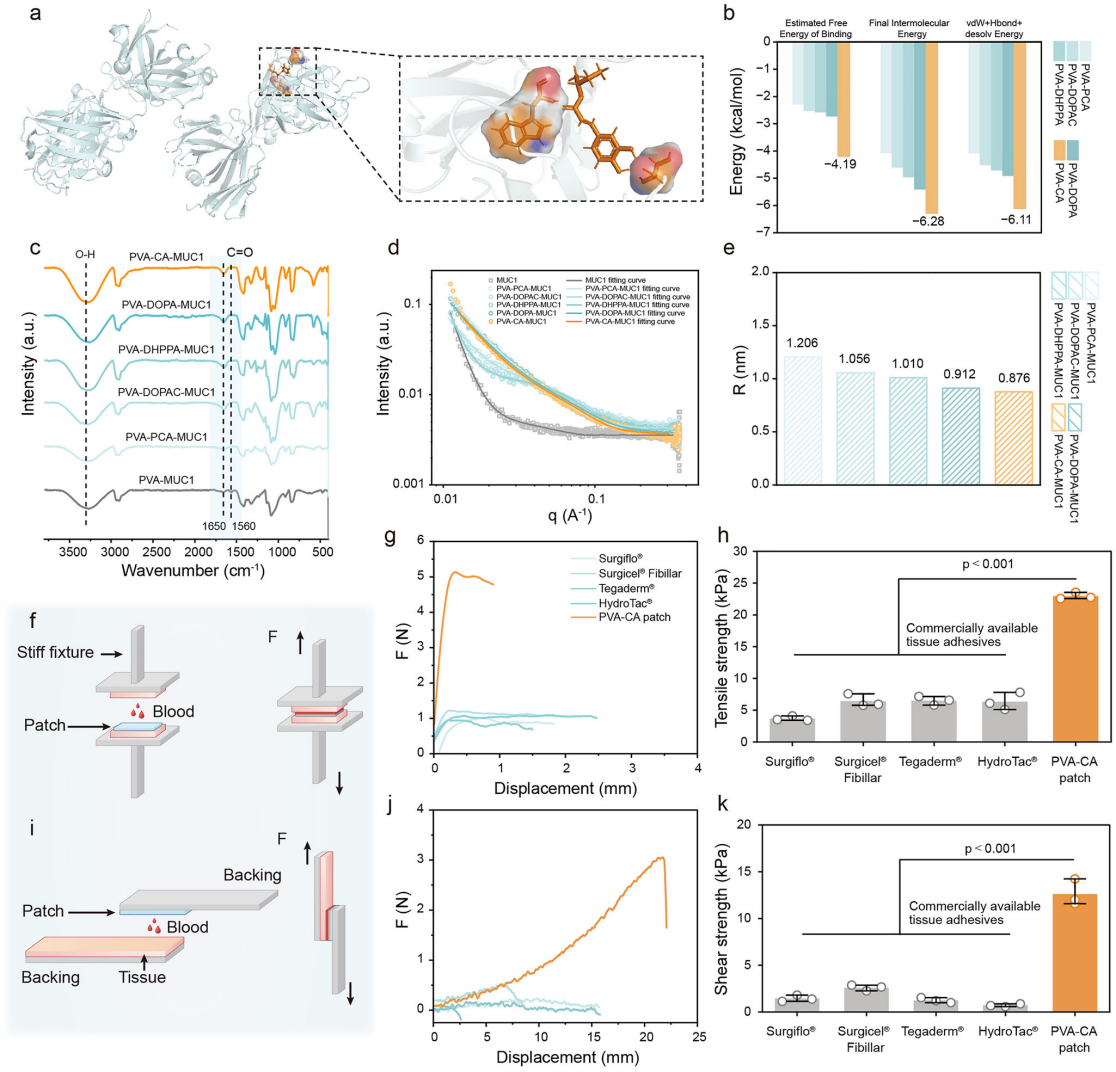
Interactions of catechol based bioahesive with tissues. (a) 3D schematic diagram of the interaction between PVA‐CA and MUC1. (b) Interaction of five adhesive systems with MUC1. (c) FTIR spectra of five adhesive systems before and after mixed with MUC1. (d) SAXS spectra of five adhesive systems before and after mixed with MUC1. (e) Phase separation correlation coefficient (R) of five adhesive systems before and after mixed with MUC1. (f) Schematic of tensile test. (g) Force‐displacement curves of the tensile test. (h) Tensile strength of Surgiflo, Surgicel Fibrillar, Tegaderm, HydroTac, and PVA‐CA patch on two side of procine gastric mucosa (n = 3 independent samples). (i) Schematic illustrating the lap shear test used for assessing the shear strength. (j) Force‐displacement curves of the lap shear test. (k) Shear strength of Surgiflo, Surgicel Fibrillar, Tegaderm, HydroTac, and PVA‐CA patch on one side of procine gastric mucosa (n = 3 independent samples). Error bars, mean ± SD. P values are determined via one‐way ANOVA followed by Tukey's post‐hoc test for h and k.

In the aforementioned study, we systematically evaluated the adhesion performance of five adhesive systems by analyzing both their cohesive and adhesive forces. To further elucidate the underlying adhesion mechanism between the adhesives and biological tissues, we conducted molecular docking studies. MUC1 mucin, one of the most broadly expressed mucins in humans, is widely distributed across epithelial tissues, particularly those lining body cavities or exposed to the external environment. We selected the characteristic VNTD domain of MUC1 (PDB ID: 8P6I) as the receptor model for molecular docking to investigate its binding interactions with the catechol‐functionalized hydrogels [56, 57]. In the 3D schematic representation, the interacting residues are illustrated using ball‐and‐stick models, while the protein backbone is shown in a cartoon format (Figure 6a; Figure S15a–d). The 2D interaction diagrams (Figure S15e) further highlight key amino acid residues, color‐coded according to their chemical properties. The results show that all five adhesive systems interact with MUC1 primarily through hydrogen bonding and non‐covalent intermolecular forces. Among them, PVA‐CA exhibited the highest binding affinity, with a binding free energy of −4.19 kcal/mol (Figure 6b). Specifically, hydrogen bonds were formed between residue TRP112 on chain H, residue SER62 on chain I of MUC1 and PVA‐CA. Additionally, van der Waals interactions (or hydrophobic effects) contributed an interaction energy of −6.11 kcal/mol, which was significantly higher than those observed for the other adhesives. This strong binding affinity suggests that PVA‐CA can establish robust interfacial interactions with mucins such as MUC1, which are abundantly present on tissue surfaces.

To further elucidate the adhesion mechanism of the film, several spectral analyses were performed in an attempt to understand the molecular interactions that occur between the tissue surfaces and the catechol‐based bioadhesives. Through comparative FTIR spectral analysis, as shown in Figure 6c, the amide I band (1650 cm^−1^) and amide II band (1560 cm^−1^) of MUC1 all shifted characteristic in the six groups of hydrogel systems, reflecting the difference in the strength of the interaction between MUC1 and hydrogel systems. Among them, the amide I band (Δν = 12 cm^−1^) and amide II band (Δν = 8 cm^−1^) of the PVA‐CA‐MUC1 complex have the most significant red shifts compared with the original sample, indicating that there is a hydrogen bond and electrostatic interaction between the catechol derivative and MUC1. SAXS was also performed that the phase separation feature size (R) [58] continued to decrease from 1.206 to 0.876 nm, and this nanoscale structural tightening showed a significant negative correlation with the enhancement of macro bond strength. This phenomenon stems from the hydrogen bond network formed between PVA‐CA chains, which preferentially induces intramolecular folding and contraction of polymer chains to form high‐density nano‐crosslinked clusters; these pre‐contracted clusters serve as efficient cross‐linking points, significantly enhancing the inter‐chain connection efficiency in the 3D network, thereby achieving improvement in macro‐mechanical properties (Figure 6d,e).

The adhesion properties of the PVA‐CA patch when in contact with blood were assessed by tensile strength and shear strength [59]. PVA‐CA patch showed excellent adhesion to blood‐covered procine skin and procine gastric mucosa (tensile strength of 22.9 ± 0.5 kPa; shear strength of 12.6 ± 1.4 kPa with porcine mucosa, Figures 6f–k and Figure S16), and its performance significantly exceeded that of commercially available tissue adhesives such as Surgiflo, Surgicel Fibrillar, Tegaderm and HydroTac.

**FIGURE 7:**
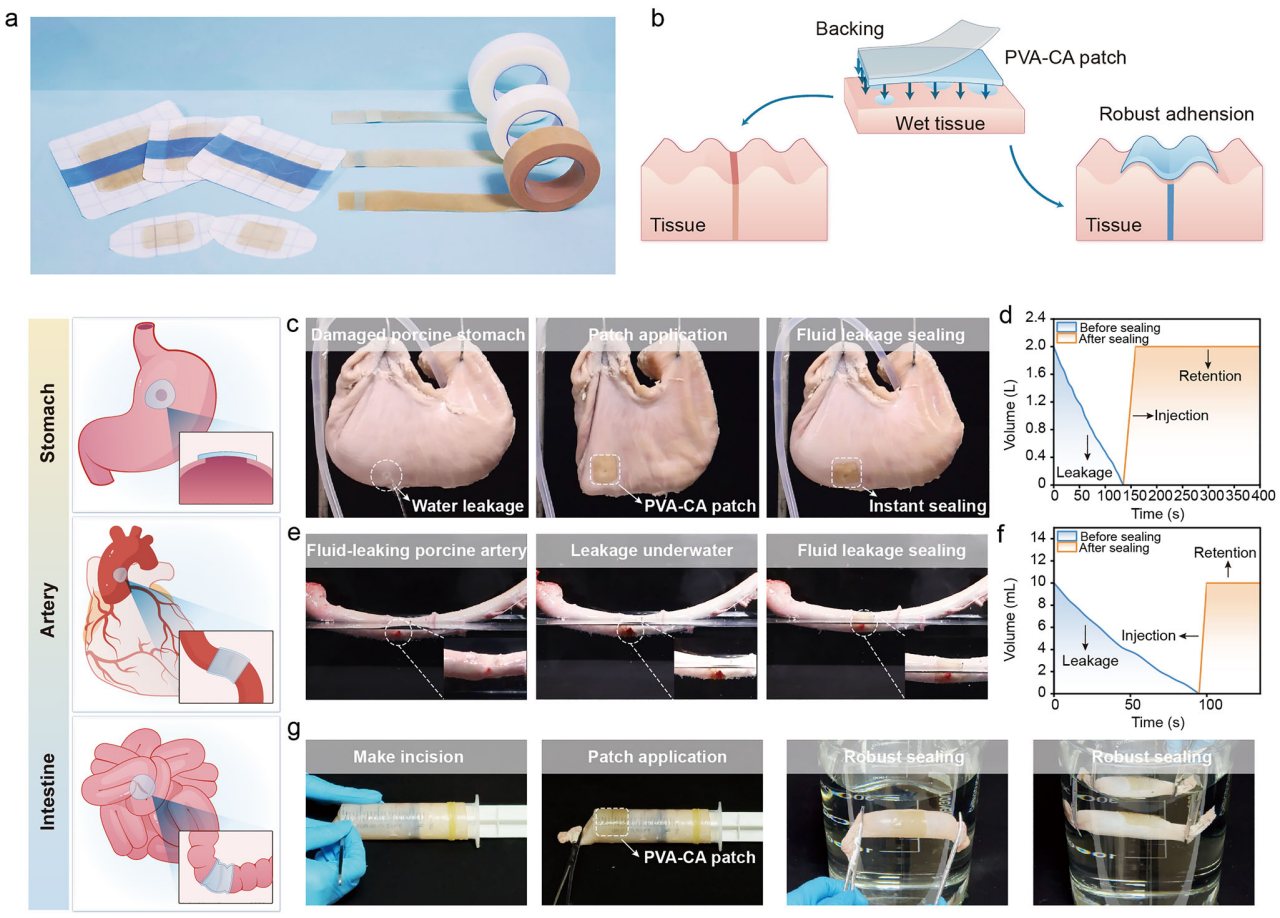
Adhesion performance of catechol‐based bioadhesives to tissues. (a) Adhesive bandages and double‐side tapes made of PVA‐CA hydrogels. (b) Schematic diagram of PVA‐CA sealing tissue gaps. Ex vivo sealing of a fluid‐leaking porcine stomach (c), a fluid‐leaking porcine artery (e) and a fluid‐leaking porcine small intestine (g) by PVA‐CA hydrogel tape. Water level in the stomach corresponding to the sealing process in (d). The amount of water in the artery corresponding to the sealing process in (f).

To evaluate the practical applicability of PVA‐CA for tissue adhesion, we performed ex vivo tests using porcine tissue models. The PVA‐CA hydrogel can be readily fabricated into various formats, including bandage‐like strips and double‐sided adhesive patches (Figure 7a), which were able to effectively seal tissue defects (Figure 7b). When applied to a gastric perforation model, the PVA‐CA patch sealed the leaking tissue within 10 s (Figure 7c,d; movie S1). The sealed stomach retained water without leakage, as confirmed by measurements of water level and volume, indicating its potential as a dressing for gastric perforation repair. To further assess tissue sealing capability, a porcine artery branch was surgically transected to simulate vascular rupture. Upon application of the PVA‐CA patch, fluid leakage ceased immediately (Figure 7e,f; movie S2), demonstrating effective sealing under physiological pressure. Finally, adhesion to intestinal tissue was examined by bonding excised segments of porcine small intestine. The PVA‐CA patch maintained strong attachment under water (Figure 7g; movie S3), underscoring its exceptional adhesive performance even under challenging conditions.

### 2.6 In vivo Evaluation of Tissue Sealing and Biocompatibility

**FIGURE 8:**
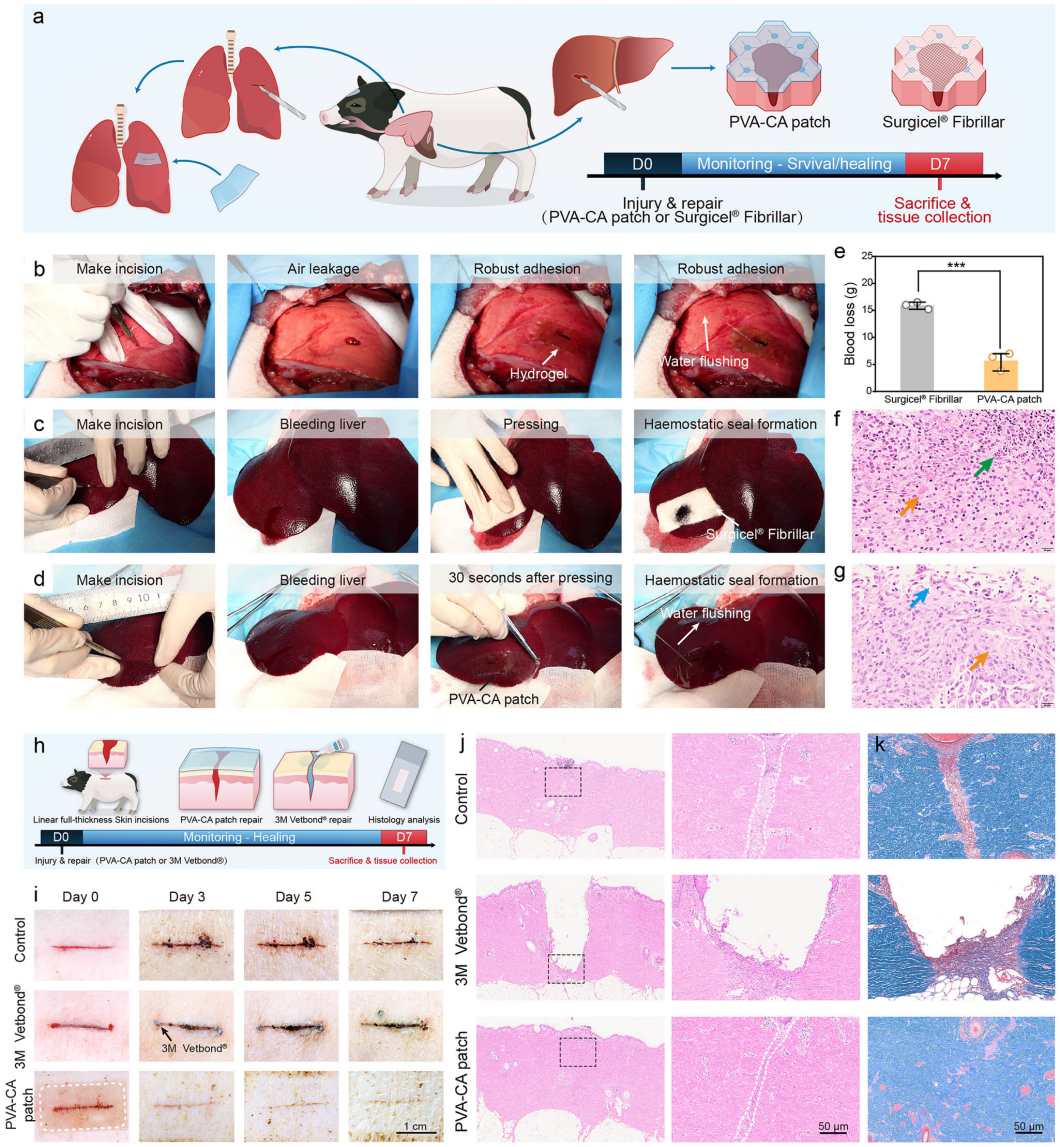
In vivo haemostatic sealing in porcine injury bleeding model. (a) Schematic illustration of the hepatic and pulmonary injury models in pigs treated by PVA‐CA patches. (b) Photographs of the hemostasis in pulmonary incision model of pig using PVA‐CA patch. (c,d) Hemostasis treatment in a porcine liver hemorrhage model. A 15‐mm long incision was made on the pig liver to establish a porcine liver hemorrhage model. Surgicel Fibrillar and PVA‐CA patches were applied to the wound and pressed manually until hemostasis was stopped. (e) Blood loss following treatment of Surgicel Fibrillar, and PVA‐CA patch in the porcine liver bleeding model (n = 3 independent injuries, ***p < 0.001, Student's *t*‐test). Representative histology images stained with H&E for Surgicel Fibrillar (f) and PVA‐CA patch (g) at1 weeks after haemostatic treatment. Yellow arrows indicate fibrous hyperplasia, blue arrows indicate inflammatory cell infiltration, and green arrows indicate necrotic liver cells. (h) Treatment flow chart of linear wounds on porcine skin. (i) Photographs of linear wounds on porcine skin, PVA‐CA patch naturally fell off on the third day. (j) H&E staining and Masone staining k) of skin wounds 1 week later. (n = 3 per group).

The ability of PVA‐CA patches to rapidly form strong and stable adhesion to biological tissues offers significant potential for rapid sealing in a variety of clinical and biomedical scenarios. To assess this capability, we evaluated the hemostatic and sealing performance of PVA‐CA patches in in vivo models of liver and lung injury in Beagle dogs and Bama minipigs (Figure 8a), as well as skin wound closure in pigs. Biocompatibility was also examined using in vivo liver models.

To validate the hemostatic and sealing efficacy on internal organs, liver and lung injury models were established in Beagle dogs (8‐10 kg), and lung injury models in Bama minipigs. A standardized incision (20 mm long × 5 mm deep) was made in the liver of Beagle dogs. The PVA‐CA patch effectively arrested bleeding and established robust interfacial adhesion at the wound site (Figure S17a and movie S8). Similarly, when applied to incisions in pulmonary lobes, the PVA‐CA patch achieved rapid sealing in both Beagle dogs and Bama minipigs. Despite the continuous expansion and contraction of lung tissue during respiration, no air leakage was observed (Figure 8b; Figure S17b and movies S4 and S7). Remarkably, PVA‐CA patches also adhered conformally and securely to the beating heart (100–120 bpm), maintaining seal integrity throughout dynamic cardiac motion (Figure S17c; movie S9).

To further demonstrate in vivo efficacy and biosafety in clinically relevant settings, we tested the performance of PVA‐CA patches for sealing hemorrhagic liver injuries in Bama minipigs, using Surgicel Fibrillar (Ethicon) as a clinical comparator. Six liver injuries (15 mm diameter × 5 mm depth) were created across pigs. Upon application, Surgicel Fibrillar became saturated due to accumulation of subwound blood (Figure 8c; movie S6), whereas PVA‐CA patches achieved complete hemostatic sealing within 30 s (Figure 8d; movie S5) and significantly reduced blood loss (Figure 8e). Both animals survived the 7‐day postoperative period without any adverse effects. Histological examination of liver tissues showed that fibrous tissue proliferation and inflammatory cell infiltration at the wound were seen in both the PVA‐CA patch group and the Surgicel Fibrillar group 7 days later, but the degree and depth of the lesion in the Surgicel Fibrillar group were significantly higher than those in the PVA‐CA patch group. In the PVA‐CA patch group, fibrous tissue at the liver wound area had mild or slight hyperplasia, and the hyperplasia was concentrated on the superficial layer of the wound surface and did not involve the inner side of the liver (Figure 8f; Figure S18a,b). In the Surgicel Fibrillar group, mild hyperplasia of fibrous tissue was seen at the wounds, accompanied by mild liver cell necrosis and inflammatory cell infiltration, and the fibrous hyperplasia spread to the medial portal area of the liver, accompanied by mild necrosis and inflammatory cell infiltration. The formation of new small bile ducts was observed in the hyperplasia fibers in the portal area. The boundary between the fibrous hyperplasia area and the normal liver lobule was unclear. The hepatocytes were vacuolated, and the liver cords were disorganized (Figure 8g; Figure S18c,d).

We also evaluated PVA‐CA patch performance in a porcine model of linear skin wound closure (Figure 8h). The patches adhered firmly to incision sites and arrested bleeding within minutes. Compared to untreated wounds and those treated with 3M Vetbond, PVA‐CA‐treated wounds exhibited improved healing outcomes (Figure 8i). Histological analysis on Day 7 revealed large epithelial gaps, hyperkeratosis, and pronounced inflammatory cell infiltration in 3M Vetbond‐treated wounds (Figures 8j–k; Figure S19), while untreated wounds exhibited persistent hyperkeratosis, periwound fibrosis, and inflammation—indicating incomplete healing. In contrast, wounds treated with PVA‐CA patches displayed minimal inflammation and significantly reduced fibrotic tissue formation around the incision sites, suggesting more favorable wound healing progression. To evaluate systemic biocompatibility, major organs (heart, liver, spleen, lungs, kidneys) were harvested and subjected to hematoxylin and eosin (H&E) staining. No signs of significant apoptosis or pathological abnormalities were observed (Figure S20), supporting the in vivo biosafety of the PVA‐CA hydrogel patches.

## 3 Conclusion

In this study, we systematically designed, synthesized, and evaluated a series of catechol‐functionalized bioadhesive hydrogels by grafting five structurally distinct catechol derivatives onto PVA via esterification.

Through a comprehensive multiscale investigation, ranging from molecular modeling and physicochemical characterization to mechanical testing and biological evaluation, we demonstrated that para‐position molecular chains could regulate hydrogel cohesion and adhesion by influencing hydrogen bonding, nanostructure, binding energy, steric effects, and related interactions. Among the candidates, the PVA‐CA hydrogel, derived from caffeic acid, exhibited the highest binding energy, crosslinking density, crystallinity, interchain hydrogen bonding, and both cohesive and adhesive strength across diverse substrates, including biological tissues. Its superior performance was further validated in ex vivo and in vivo models, where PVA‐CA patches achieved rapid and robust tissue sealing, hemostasis, and biocompatibility. Overall, this work underscores the potential role of para‐position molecular design in optimizing hydrogel adhesives for next‐generation wet‐tissue adhesives with broad biomedical potential.

Despite these promising findings, certain limitations remain. For instance, while the PVA‐CA hydrogel exhibited excellent cohesive strength, excessive cohesion may increase the risk of interfacial adhesive failure under dynamic physiological conditions. Additionally, long‐term in vivo degradation behavior, immune responses, and integration with regenerating tissues warrant further exploration to fully establish clinical applicability. Future research should focus on balancing cohesive and adhesive forces to maximize interfacial stability under complex in vivo environments. Moreover, tailoring the chemical structure of catechol derivatives or incorporating stimuli‐responsive functionalities may enable the development of next‐generation smart bioadhesives with controllable adhesion, biodegradability, and regenerative potential. This work provides a robust structural and mechanistic framework for the rational design of high‐performance bioadhesives with broad translational potential in tissue engineering and wound management.

## 4 Experimental Section

### 4.1 Synthesis and Characterization of Catechol‐Based Bioadhesives

The synthesis of catechol‐based adhesives was detailed in the supporting information. Five catechol‐based bioadhesives were characterized by ^1^H NMR, UV‐visible spectroscopy, FT‐IR and viscometry. Details of characteristic processes and results are found in the SI.

### 4.2 Adhesion Testing

Sample preparation: Rigid substrates (stainless steel, aluminum, glass fiber, plastic, acrylic, resin, synthetic stone, bovine bone) were cleaned sequentially with deionized water and ethanol, then air‐dried. Soft biological substrates (porcine skin and procine gastric mucosa) were rinsed with deionized water to remove surface lipids and contaminants. Refer to SI for the detailed process of lap‐shear adhesion test, tensile adhesion test and peel adhesion test.

### 4.3 Characteristics of Cohesion and Adhesion Force of Catechol‐Based Bioadhesives

The cohesion and adhesion of catechol‐based adhesives were compared by AFM, SAXS, WAXS and GIWAXS. Details of characteristic processes and results are found in the SI.

### 4.4 In Vitro and in Vivo Adhesion Properties of PVA‐CA Patch

In vitro gastric perforation, arterial leak and intestinal perforation models were used to verify adhesion properties of PVA‐CA patches. In vivo adhesion properties and biocompatibility of PVA‐CA patches were verified using Bama pig and Beagle dog animal experiments. Details of characteristic processes and results are found in the SI.

### 4.5 Ethical Statement

All the in vivo experiments had been approved (LLSN‐2025194; LLSN‐2025202) by the Ethics Committee of Sichuan Lilaisinuo Biological Technology Co.

## Author Contributions

The manuscript was written through the contributions of all authors. All authors have given approval to the final version of the manuscript.

## Conflicts of Interest

The authors declare no conflict of interest.

## Supporting information




**Supporting File 1**: advs73621‐sup‐0001‐SuppMat.docx.

**Supporting File 2**: advs73622‐sup‐0002‐MovieS1.mp4.

**Supporting File 3**: advs73623‐sup‐0002‐MovieS2.mp4.

**Supporting File 4**: advs73624‐sup‐0002‐MovieS3.mp4.

**Supporting File 5**: advs73625‐sup‐0002‐MovieS4.mp4.

**Supporting File 6**: advs73626‐sup‐0002‐MovieS5.mp4.

**Supporting File 7**: advs73627‐sup‐0002‐MovieS6.mp4.

**Supporting File 8**: advs73628‐sup‐0002‐MovieS7.mp4.

**Supporting File 9**: advs73629‐sup‐0002‐MovieS8.mp4.

**Supporting File 10**: advs736210‐sup‐0002‐MovieS9.mp4.

## Data Availability

The data that support the findings of this study are available from the corresponding author upon reasonable request.
