# Supplementary material for: Catechol Derivative‐Based Bioadhesives: Molecular Design for Precision Medical Adhesion
Source: Adv Sci (Weinh). 2026 Jan 7;13(27):e21272. doi: 10.1002/advs.202521272 (PMC13170217; doi:10.1002/advs.202521272)
Supplement: Supplementary file 1 — Supporting File 1: advs73621‐sup‐0001‐SuppMat.docx. [file ADVS-13-e21272-s004.docx]

Supporting Information

**Catechol Derivative-Based Bioadhesives: Molecular Design for Precision Medical Adhesion**

Xueyu Wang , Zixin Yang , Shan Wang , Weiwei Tan , Zhirui He , Qinyu Duan , Huanan Wang , Tao Chen*, and Shanshan Hu*

X. Wang, Z. Yang, S. Wang, W. Tan, Z. He, Q. Duan, Prof. Dr. T. Chen and Prof. Dr. S. Hu

Chongqing Key Laboratory of Oral Diseases, Chongqing Municipal Key Laboratory of Oral Biomedical Engineering of Higher Education, Chongqing Municipal Health Commission Key Laboratory of Oral  Biomedical  Engineering, The Affiliated Stomatological Hospital of Chongqing Medical University, Chongqing, 401147, P. R. China

E-mail: chentao1985@hospital.cqmu.edu.cn

hushanshan@hospital.cqmu.edu.cn

Prof. Dr. H. Wang

Key State Laboratory of Fine Chemicals, School of Bioengineering Dalian University of Technology, Dalian, 116023, P. R. China

Xueyu Wang and Zixin Yang contributed equally to this work.

**Table of Contents**

1. **Experimental processes and characterizations** 3
   1. **Materials** 3

**1.2 Synthesis of catechol-based bioadhesives**3

**1.3 Characterization of catechol-based bioadhesives**3

**1.4 Density functional theory (DFT) calculations**4

**1.5 Swelling Ratio Test**4

**1.6 Lap-shear adhesion test**4

**1.7 Tensile adhesion test**4

**1.8 Peel adhesion energy test**5

**1.9 Atomic Force Microscopy (AFM) Measurements**5

**1.10 Small- and Wide- Angle X-ray Scattering (SAXS and WAXS)**5

**1.11 Grazing-incidence wide-angle X-ray scattering (GIWAXS)**6

**1.12 Molecular Docking**6

**1.13 Ex Vivo Tissue Adhesion Assay**6

**1.14 Exploration of the interactions between the catechol based bioahesive and tissues**6

**1.15 In Vivo Beagle Dog Models**7

**1.16 In Vivo Porcine Liver Injury and Pulmonary Air Leak Models**7

**1.17 In Vivo Porcine Skin Wound Closure Models**8

**1.18 Statistical analysis**8

- 1. **Ethical Statement**8

1. **Supplementary Figures** 9
2. **Supplementary Tables** 29
3. **Legends for movies S1 to S9** 30
4. **Supplementary References**31
5. **Experimental processes and characterizations**

**1.1 Materials**

Poly(vinyl alcohol) (PVA, Mw 85-124 kDa) was purchased from Sigma-Aldrich (USA). Dimethyl sulfoxide (DMSO) and sodium bisulfate monohydrate (NaHSO₄·H₂O) were obtained from J&K Scientific (USA). Catechol derivatives, including 3,4-dihydroxybenzoic acid (PCA), 3,4-dihydroxyphenylacetic acid (DOPAC), 3,4-dihydroxyhydrocinnamic acid (DHPPA), 3,4-dihydroxy-D-phenylalanine (DOPA), and 3,4-dihydroxycinnamic acid (CA), were purchased from Aladdin (Shanghai, China). Surgiflo^®^ (Ethicon, USA), Tegaderm^®^ (3M, USA), HydroTac^®^(HARTMANN, Germany), Surgicel^®^ Fibrillar (Ethicon, USA) and Vetbond^®^ (3M, USA) were obtained from commercial sources.

**1.2 Synthesis of** **catechol-based bioadhesives**

The five catechol-based adhesives were synthesized following protocols from our previous work. Briefly, PVA (48 mmol) was dissolved in DMSO (120 mL) at 100 °C. After complete dissolution, NaHSO₄·H₂O (6 g) was added, and the temperature was reduced to 80 °C. Subsequently, 12 mmol of each catechol derivative (PCA, DOPAC, DHPPA, DOPA, or CA) was added to the solution. The reaction proceeded at 80 °C under a nitrogen (N₂) atmosphere for 24 hours. The reaction mixture was then purified by dialysis (MWCO 3,500 Da; Solarbio, China) against deionized water for 7 days and lyophilized under vacuum. The lyophilized products were redissolved in ultrapure water at a concentration of 200 mg/mL to yield the hydrogel precursors: PVA-PCA, PVA-DOPAC, PVA-DHPPA, PVA-DOPA, and PVA-CA. Hydrogel films were fabricated by casting the 200 mg/mL solutions into silicone molds and curing under vacuum at 37 °C.

**1.3 Characterization of catechol-based bioadhesives**

The chemical structures of the synthesized catechol-modified polymers were confirmed by Fourier transform infrared spectroscopy (FTIR, Nicolet iS20, Thermo Fisher Scientific, USA), ultraviolet–visible spectroscopy (UV-Vis, UV-2500, Shimadzu, Japan), and proton nuclear magnetic resonance (1H NMR, Bruker 400 MHz, Germany). Surface morphology of hydrogel films was examined using scanning electron microscopy (SEM, TESCAN MIRA LMS, Czech Republic). Rheological properties were characterized using a rotational rheometer (MCR 302, Anton Paar, Germany) at 25 °C. A 40 mm parallel-plate geometry with a 1 mm gap was employed. Frequency sweep tests were performed at 1% strain amplitude over an angular frequency range of 0.1-100 rad/s to determine the storage (G′) and loss (G″) moduli.

**1.4 Density functional theory (DFT) calculations**

All DFT calculations were performed with Gaussian 16, Rev. A03 programs. The B3LYP hybrid functional^[1]^ including Grimme empirical dispersion correction (D3BJ)^[2]^ with 6-31G(d) basis set were used for the geometry optimization of all structures. Harmonic vibrational frequency analysis was calculated at the same level to verify that geometric structure has positive frequency. The B3LYP hybrid functional with def2-tzvp^[3]^ basis set were used to calculated energy of all structures. The calculations and visualizations of ESP surface were performed by Multiwfn^[4]^ and VMD^[5]^ software. The independent gradient model based on Hirshfeld partition of the molecular density (IGMH) was adopted in the visual analysis of the intermolecular interactions of the optimized PVA-PCA, PVA-DOPAC, PVA-DHPPA, PVA-DOPA, and PVA-CA which was performed on Multiwfn software. The weak interaction regions were visualized with a Sign(*λ_2_*)r colored isosurfaces isovalue of 0.005 a.u.

**1.5 Swelling Ratio Test**

To evaluate the swelling ratio, testing hydrogel samples (measuring 17 mm in diameter and 1 mm in thickness) were immersed in PBS at a temperature of 37 °C. The samples were retrieved and weighed using precise balances (W) at the predetermined time. The swelling ratio (SR %) was then determined as follows

$$SR \%= \frac{W-W_{0}}{W_{0}} \times100\%$$

W_0_ is the initial weight of the hydrogel sample.

**1.6 Lap-shear adhesion test**

Substrates were cut into standardized dimensions: 100 mm × 25 mm × 2 mm for lap-shear tests. Hydrogel (50 μL) was applied at the bonding interface, and the samples were joined in a lap-shear configuration. After curing for 12 hours, adhesive strength was measured using a universal testing machine (Criterion 43, MTS Systems, USA) at a crosshead speed of 0.1 mm/min. The maximum stress prior to failure was recorded.

**1.7 Tensile adhesion test**

Hydrogel patches (15 mm × 15 mm) were prehydrated in ultrapure water for 10 seconds before application. Two substrates were bonded in an end-to-end configuration using the patch. After 12 hours of curing, tensile adhesive strength was measured using the same testing machine. The peak stress before bond failure was recorded.

**1.8 Peel adhesion energy test**

Porcine skin and oral mucosa were cut into standardized samples (75 mm × 15 mm × 2 mm). A 100 μm-thick polyester film was adhered to the tissue surface using cyanoacrylate adhesive (Krazy Glue^®^) for mechanical reinforcement. A 180° peel test was performed at a constant speed of 15 mm/min using the universal testing machine. The free end of the substrate was clamped. The peel force per unit width (F/w) was recorded, and the adhesion energy (Γ) was calculated as 2 × F/w.

All adhesion experiments (lap-shear, tensile, and peel tests) were conducted using at least five replicates (n ≥ 5) for statistical reliability.

**1.9 Atomic Force Microscopy (AFM) Measurements**

The surface morphology of the catechol-based adhesives was characterized using atomic force microscopy (AFM; Dimension Icon, Bruker Corporation, USA) in tapping mode. A silicon cantilever with a sharp triangular pyramid tip (OLTESPA-R4, Bruker Corporation) was used for imaging. The scan area was set to 1.6 × 1.6 μm². NanoScope Analysis software (version 1.8, Bruker Corporation) was used to calculate the average root mean square (RMS) surface roughness. Surface roughness values were averaged from three independent samples to ensure reproducibility. AFM-based force measurements were also conducted using the same instrument and cantilever under tapping mode at room temperature. In a typical experiment, the AFM tip was approached perpendicularly toward the hydrogel-coated mica substrate until it made contact and slightly penetrated the surface, followed by retraction. Adhesive interactions were identified as negative force values on the retraction curve. The force-distance curves were analyzed using NanoScope Analysis software (version 1.8, Bruker Corporation) to determine adhesive force values.

**1.10 Small and Wide Angle X-ray Scattering (SAXS and WAXS)**

Two-dimensional (2D) small-angle X-ray scattering (SAXS) and wide-angle X-ray scattering (WAXS) measurements were performed using a Xeuss 3.0 SAXS/WAXS system. For SAXS, an X-ray beam with a wavelength of 1.03 Å was used. Scattering patterns were collected using a Pilatus 1M detector with an exposure time of 10 s. To enhance signal intensity, five swollen PVA-based hydrogel films were stacked for SAXS measurements. For WAXS, measurements were performed on dried films using Cu Kα radiation (λ = 1.54 Å) as the incident beam. A 2D scattering pattern was recorded with an Eiger2R 1M detector at an exposure time of 1200 s. The degree of crystallinity (χc) was calculated using multi-peak Gaussian fitting based on the following equation:

$$\chi c=\frac{\sum\mathrm{Ac}}{\sum\mathrm{Ac}+\sum\mathrm{Aa}}\times100\%$$

where Ac and Aa are the integrated areas of the crystalline and amorphous domains, respectively. The fitting and area integration were performed using standard data processing protocols to ensure consistency and accuracy.

**1.11 Grazing-incidence wide-angle X-ray scattering (GIWAXS)**

Grazing incidence wide-angle X-ray scattering (GIWAXS) measurements were carried out at the BL02U2 beamline of the Shanghai Synchrotron Radiation Facility (SSRF). The X-ray wavelength was 1.24 Å, and the grazing incidence angle was 0.2°with respect to the substrate.

**1.12 Molecular Docking**

The protein structure used for molecular docking was retrieved from the Protein Data Bank (PDB ID: 8P6I). Docking simulations were performed using AutoDock 4.2. The docking grid was centered at coordinates x = -17.558, y = 8.187, and z = 24.755, encompassing the predicted binding pocket. A grid box measuring 82 × 66 × 126 Å³ with a grid point spacing of 0.5 Å was defined. The Lamarckian Genetic Algorithm (LGA) was employed with a maximum of 10,000 energy evaluations. The conformation exhibiting the most favorable binding energy was selected for analysis. Two-dimensional (2D) ligand-protein interaction diagrams were generated using Discovery Studio Visualizer (BIOVIA), and three-dimensional (3D) binding poses were visualized using PyMOL (Schrödinger, LLC).

**1.13 Exploration of the interactions between the catechol based bioahesive and tissues.**

MUC1 (MedChemExpress, HY-P2508) was dissolved in PBS (1 mg/ml) and sonicated for 30 minutes. The MUC1 solution was then reacted with different hydrogel solutions at 37°C on a shaker (150 rpm). The mixture was examined by FT-IR (Nicolet iS20, Thermo Fisher Scientific, USA) and SAXS (Xenocs Xeuss 3.0, France) spectroscopy.

**1.14 Ex Vivo Tissue Adhesion Assay**

To assess the sealing capability of the PVA-CA hydrogel, ex vivo models simulating various tissue injuries were developed using porcine organs. For gastric perforation sealing, a 3-mm-diameter hole was created in a porcine stomach. A tube containing flowing water was connected to the top of the stomach to observe fluid leaks. Upon injection of 2000 mL of water, leakage through the perforation was observed. Then PVA-CA hydrogel was applied to the hole and waited for 10 seconds. After injecting water into the stomach again, the leakage stopped. The internal water level was also monitored through a connecting pipe. To simulate vascular rupture, the surrounding fluid was replaced with a 20 mg/mL vitamin C solution, and simulated blood (10 mg/mL potassium permanganate solution) was injected. A collateral branch of the porcine artery was surgically transected, resulting in immediate fluid leakage. The PVA-CA hydrogel was applied to the bleeding site. Upon reinjection of the potassium permanganate solution, no further leakage was observed. For intestinal anastomosis sealing, the porcine small intestine was transected and rejoined using a PVA-CA patch. Uniform pressure was applied along the patch edges for 10 seconds. Air was then injected into the intestinal cavity and placed under water to verify the sealing efficacy.

**1.15 In Vivo Beagle Dog Models**

Male Beagle dogs (12 months old, 12-15 kg; Chengdu Lilai Biotechnology Co., Ltd.) were used to establish in vivo liver and lung injury models. All surgical procedures were performed under general anesthesia maintained by intravenous infusion of propofol (0.1-0.2 mg/kg/min). For the hepatic hemorrhage model, a 20-mm-long × 5-mm-deep incision was created on the liver with a scalpel and PVA-CA patches were applied to the bleeding site. The hemostatic efficacy and wound sealing effect were evaluated following manual compression for under 30 seconds.For the canine pulmonary air leak model, a 15-mm-long × 10-mm-deep incision was made on lung lobes with PVA-CA patches applied to wounds. The air-tight sealing effect was verified via saline irrigation testing after 10-second compression. Additionally, the canine heart was surgically exposed with PVA-CA patches applied directly to myocardial tissue, and adhesion integrity was assessed under saline irrigation during cardiac motion.

**1.16 In Vivo Porcine Liver Injury and Pulmonary Air Leak Models**

Bama minipigs (22-26 kg; Chengdu Lilai Biotechnology Co., Ltd.) were used to establish in vivo models of hepatic bleeding and pulmonary air leakage. General anesthesia was induced and maintained using propofol (0.1-0.2 mg/kg/min). To establish liver injury models, pre-weighed medical gauze was positioned beneath the liver before creating 15-mm-long × 5-mm-deep linear incisions on hepatic lobes using a scalpel (three independent wounds per pig; twopigs total). Free bleeding was allowed for 20 seconds (pre-treatment blood loss) to assess hemorrhage volume. Pre-existing blood was blotted with pre-weighed gauze before applying hemostatic materials. Both PVA-CA patches and Surgicel^®^ Fibrillar were directly applied to wounds with manual compression for 30 seconds. Hemostasis was evaluated after releasing pressure and each treatment group included three independent wounds. Blood absorbed by weighed gauze or hemostatic materials was recorded as post-treatment blood loss. Abdominal cavities were closed via layered suturing before pigs were recovered from anesthesia and extubated. At 1-week post-implantation, pigs were humanely euthanized. The wound sites and visceral organs were excised and fixed in 10% formalin for 24 hours for histological analysis. For porcine pulmonary air leak models, 15-mm-long × 10-mm-deep incisions were made on lung lobes, with PVA-CA patches applied to the wounds. After 10-second compression, air-tight sealing was verified through irrigation testing.

**1.17 In Vivo Porcine Skin Wound Closure Model**

Linear full-thickness skin incisions were established on the dorsal skin of Bama minipigs (22-26 kg; Chengdu Lilai Biotechnology Co., Ltd.) under general anesthesia maintained with propofol (0.1-0.2 mg/kg/min). The surgical area was shaved and sterilized with 70% ethanol. Full-thickness skin incisions (15-mm long × 8-mm deep) were created on porcine dorsums (three independent wounds per animal; three pigs total). PVA-CA patches or 3M Vetbond^®^ weas then applied to the wounds, with untreated wounds serving as controls. After 7 days, pigs were humanely euthanized and skin tissues from wound sites were harvested followed by 24-hour fixation in 10% formalin for subsequent histological analysis.

**1.18 Statistical analysis**

Data were presented as mean ± SD. In the statistical analysis for comparison between multiple samples, a one-way ANOVA followed by Tukey’s post-hoc test was conducted. In the statistical analysis between two data groups, a two-sided Student’s t-test was used. Statistical analysis was performed using GraphPad Prism software (version 9.0.0). p values less than 0.05 were considered significantly significant. *p < 0.05, **p < 0.01, ***p < 0.001, ****p < 0.0001; ns, not significant.

**1.19 Ethical Statement**

All animal experiments were performed according to protocols approved by the Ethics Committee of SiChuan Lilaisinuo Biological Technology Co. (LLSN2025194; LLSN2025202).

1. **Supplementary Figures**

**
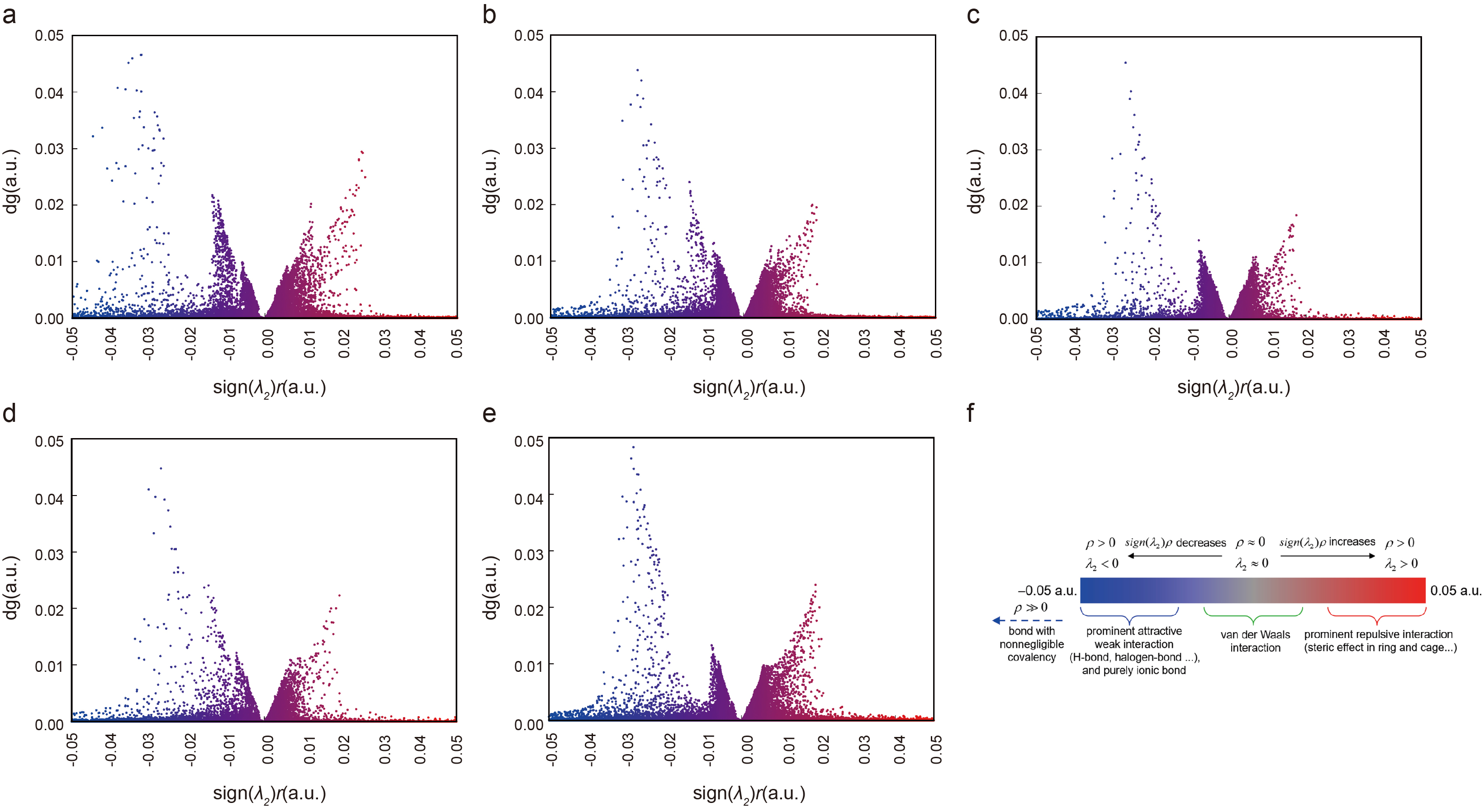
**

**Figure S1.** Sign(λ₂)ρ-colored IGMH scatter plot illustrating the interaction between chains of the five products following the esterification reaction. a) PVA-PCA, b) PVA-DOPAC, c) PVA-DHPPA, d) PVA-DOPA, e) PVA-CA.


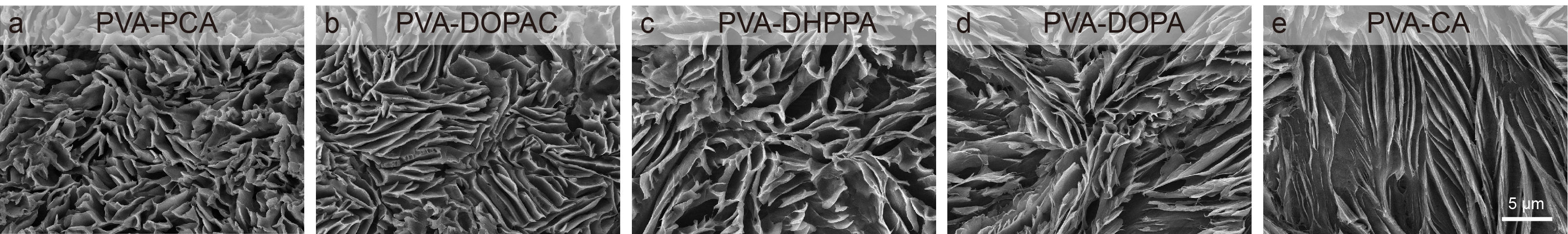


**Figure S2.**  SEM images of the five adhesive systems.


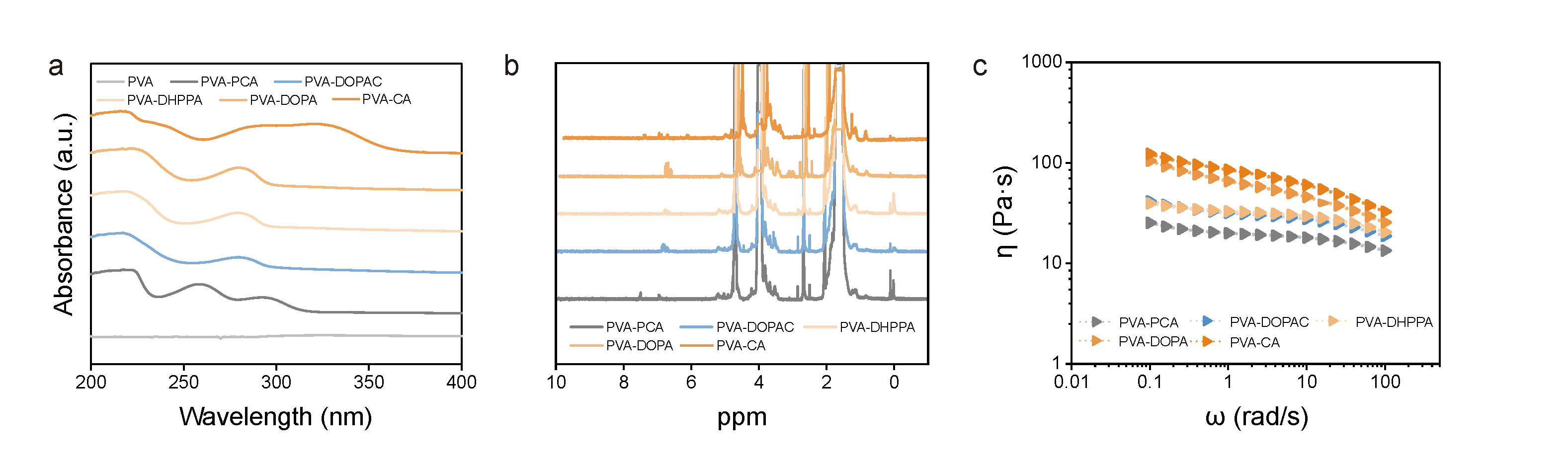


**Figure S3.** UV-Vis a) and ¹H NMR b) spectra of the five catechol-based bioadhesives.  c) Viscosity profiles of the five adhesive systems.

**
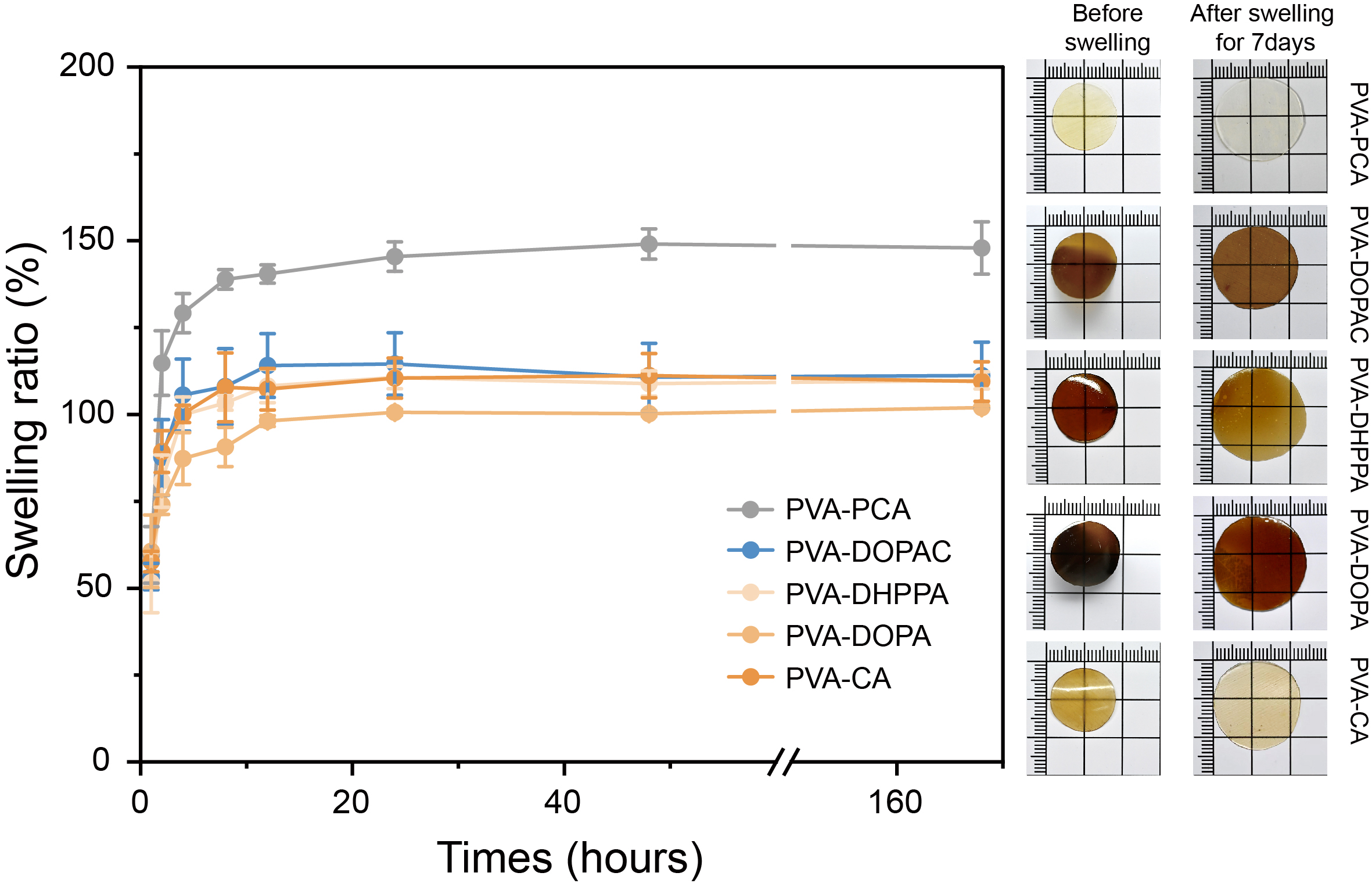
**

**Figure S4.** Swelling ratio assessment of hydrogel in PBS over 168 h.


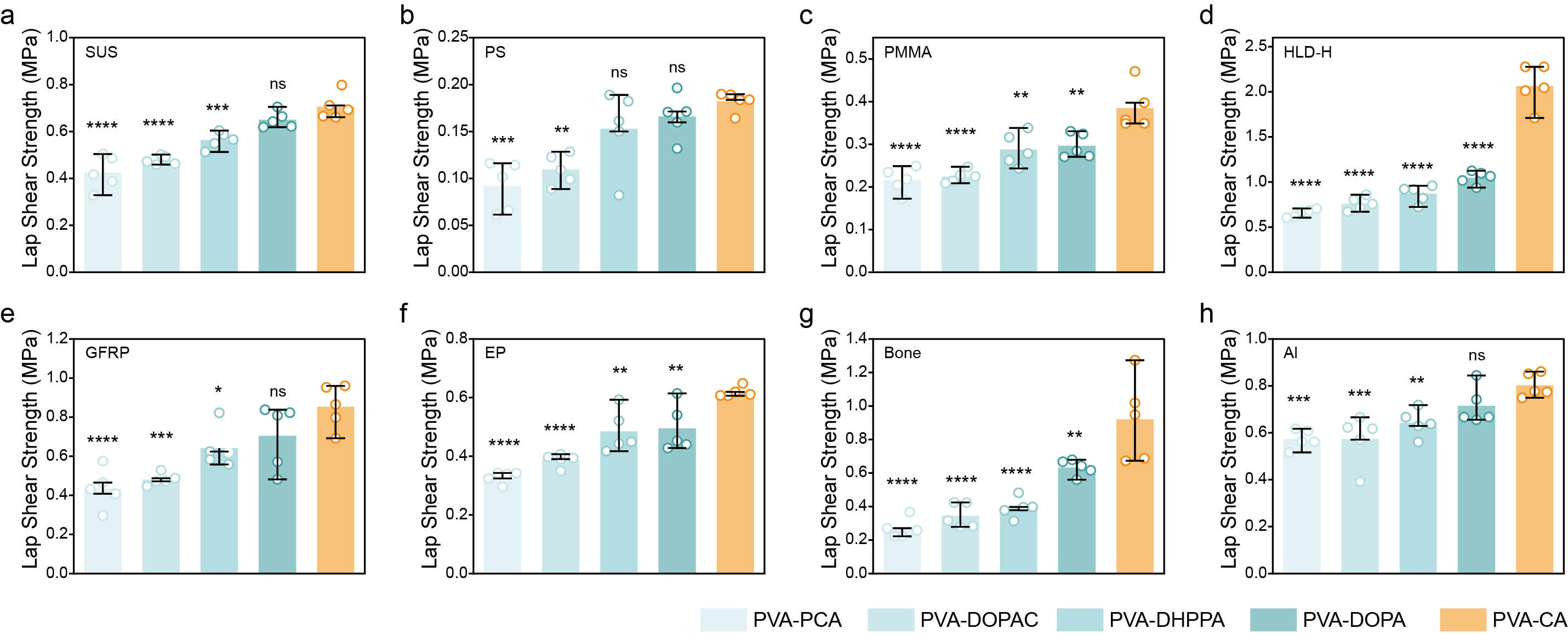


**Figure S5.** The lap shear adhesion strength for the five adhesive systems on a series of representative substrates. (n=5 per group, **p <* 0.05, ***p<* 0.01, ****p <* 0.001, *****p <* 0.0001, ns, not significant, one-way ANOVA, Tukey’s post hoc analysis).


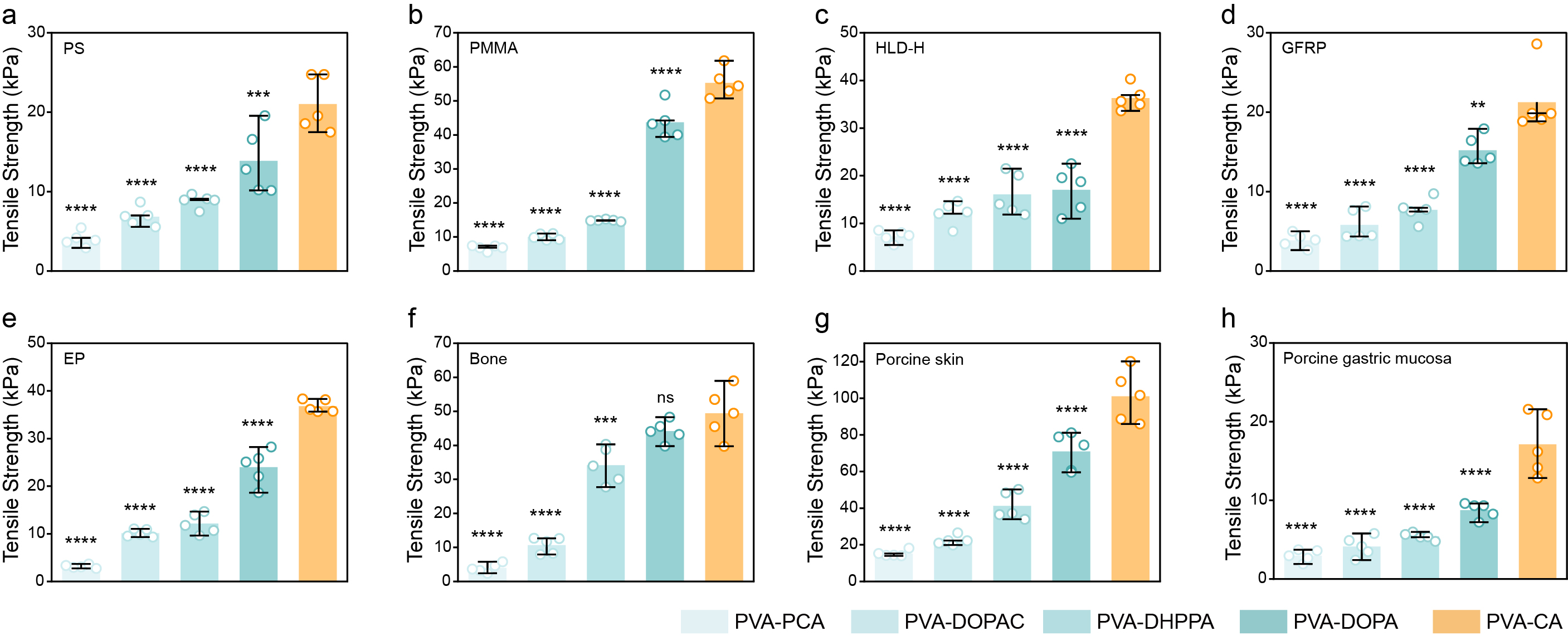


**Figure S6.** The tensile adhesive strength for the five adhesive systems on a series of representative substrates ((n=5 per group, **p <* 0.05, ***p<* 0.01, ****p <* 0.001, *****p <* 0.0001, ns, not significant, one-way ANOVA, Tukey’s post hoc analysis).


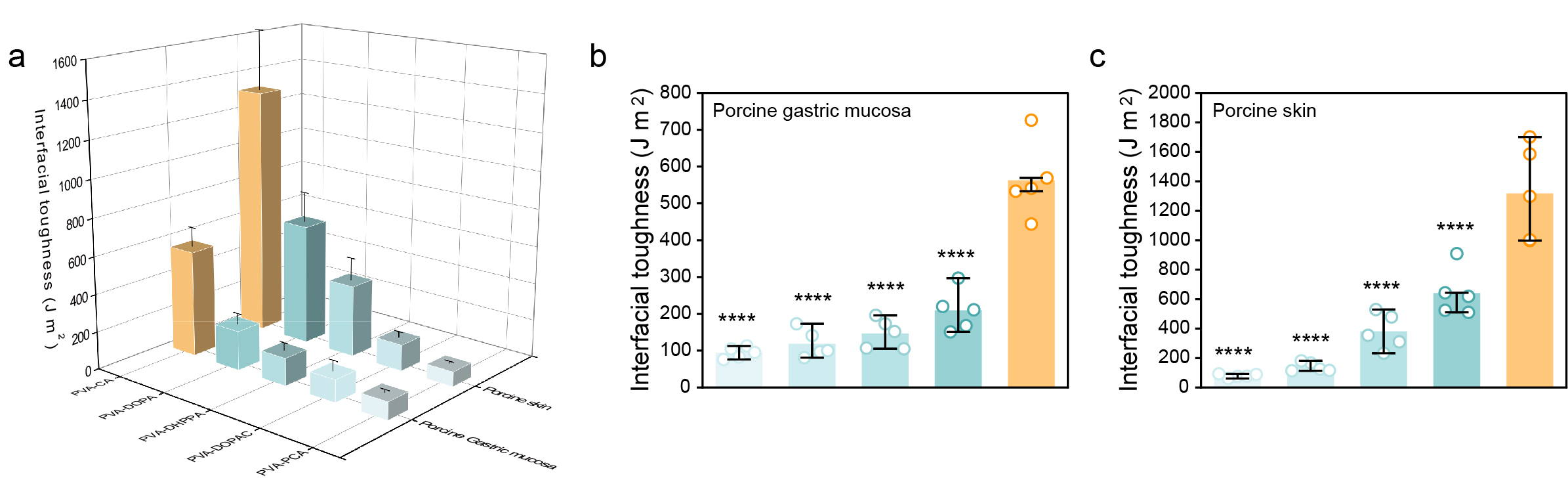


**Figure S7.** The Interfacial toughness for the five adhesive systems on procine gastric mucosa and procine skin ((n=5 per group, **p <* 0.05, ***p<* 0.01, ****p <* 0.001, *****p <* 0.0001, one-way ANOVA, Tukey’s post hoc analysis).


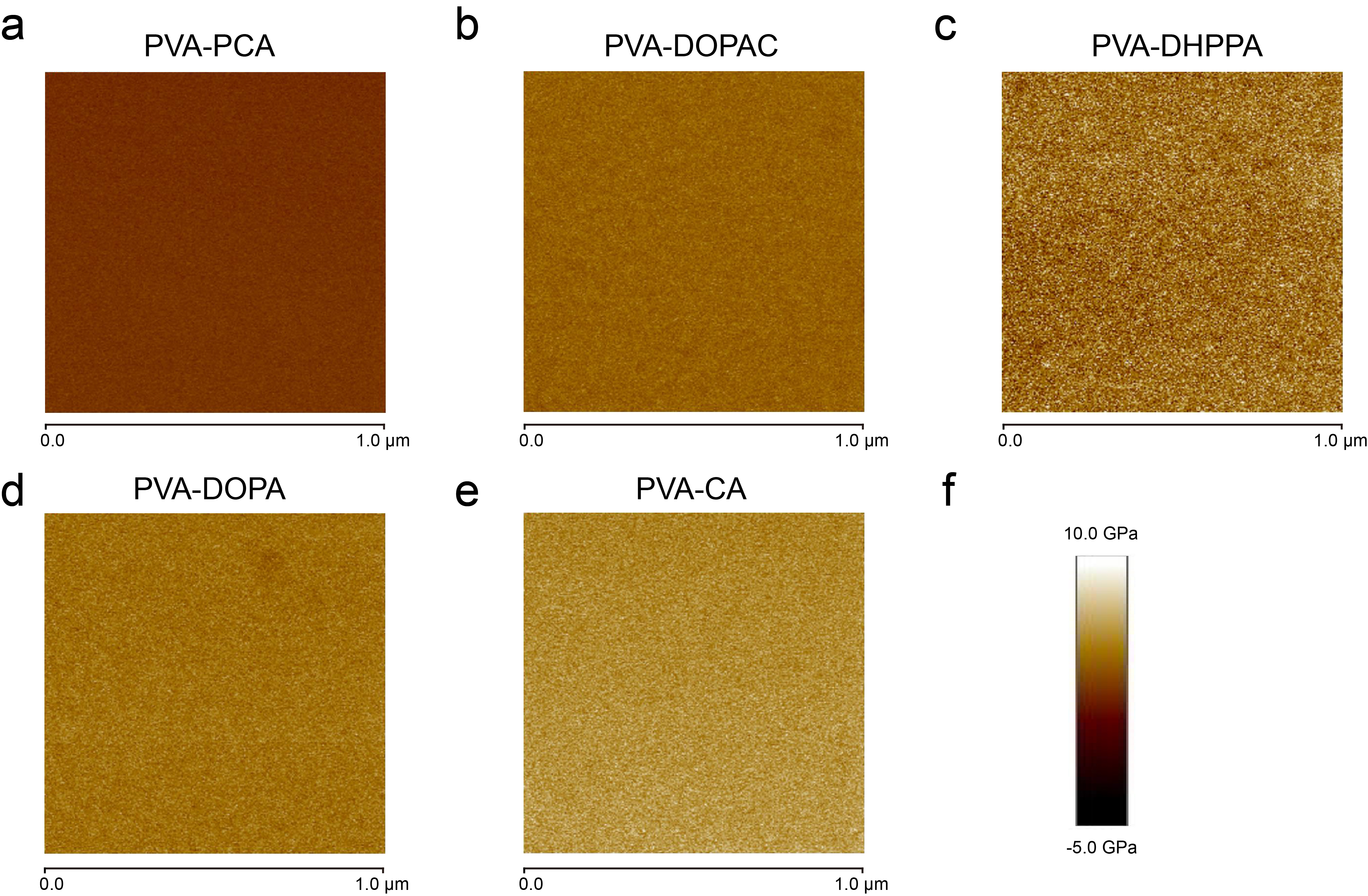


**Figure S8.** DMT modulus distribution of the five adhesive systems.


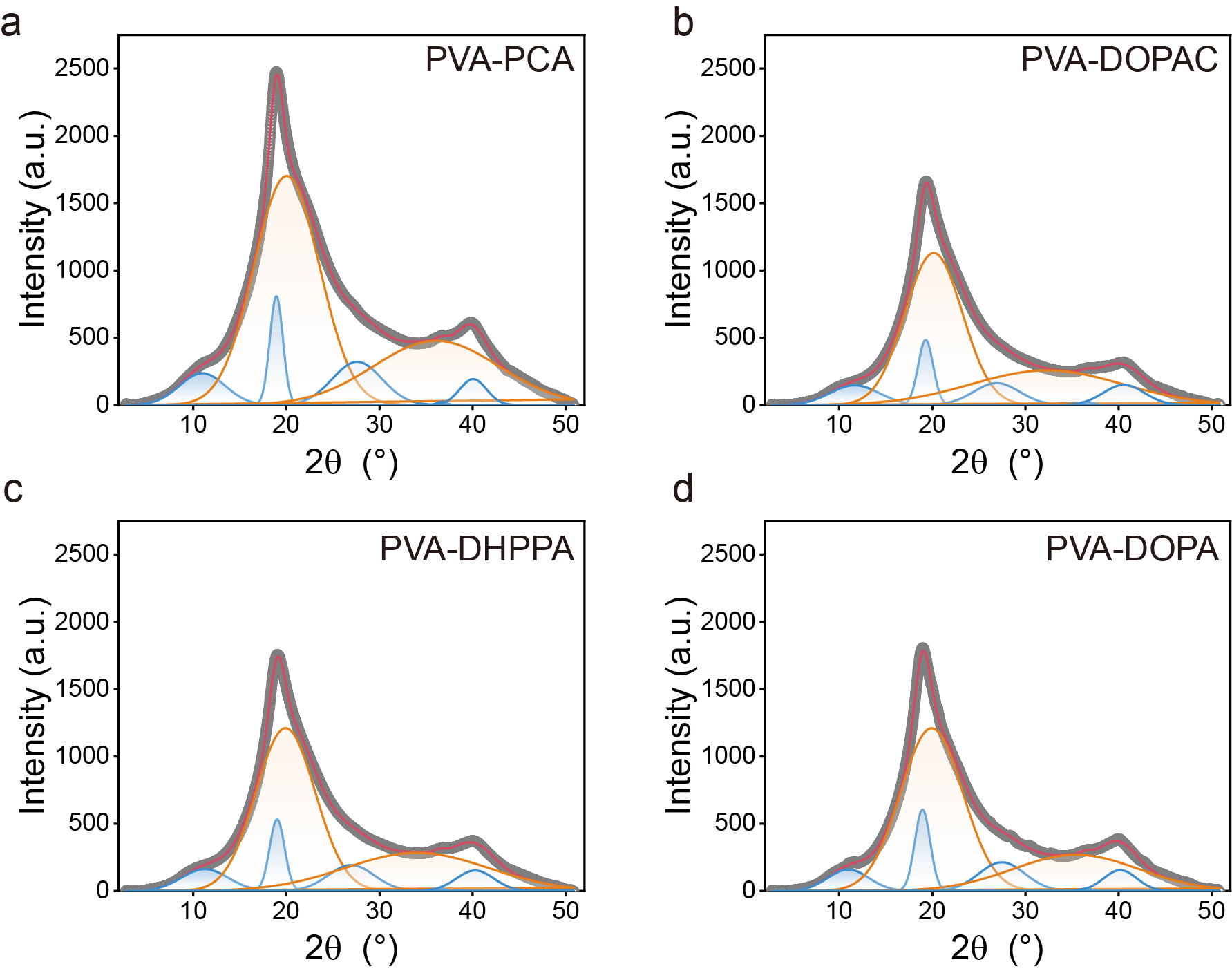


**Figure S9.** Multipeak fitting of the 1D integrated WAXS curve.

**
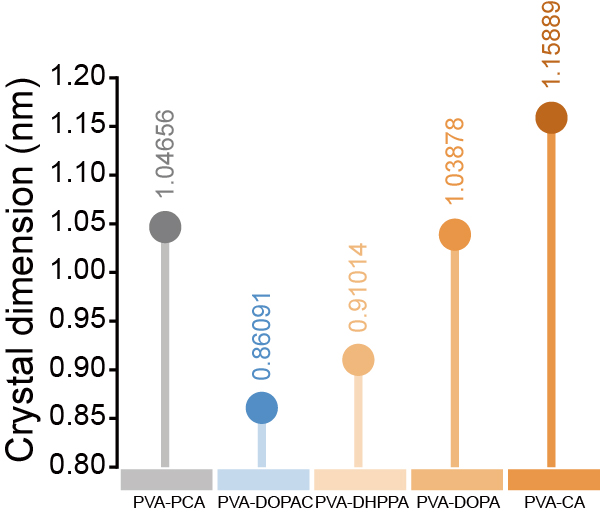
**

**Figure S10.** The dimensions of the five adhesive systems.


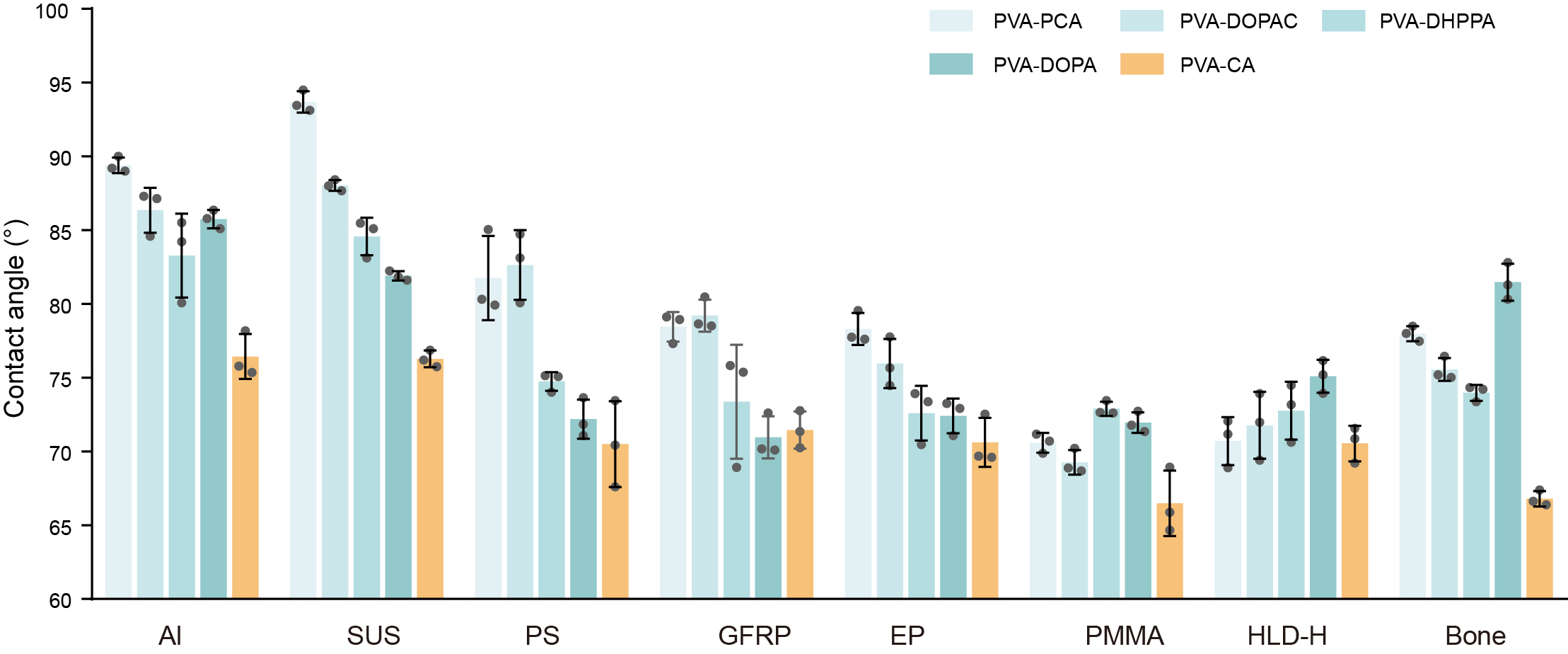


**Figure S11.** Contact angles of five catechol hydrogels at different interfaces. (n=3)


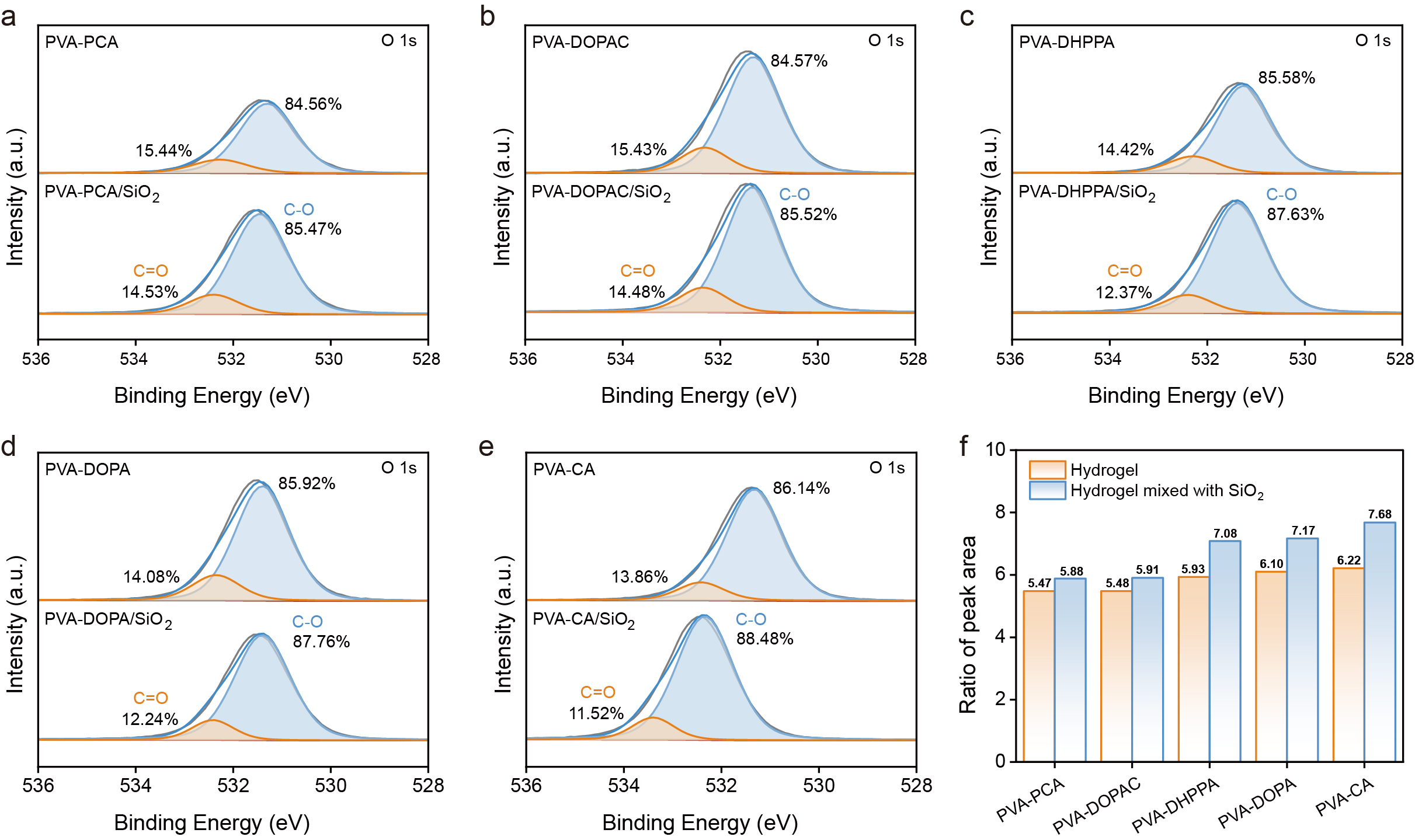


**Figure S12**. a-e)XPS spectra of five adhesive systems/SiO_2_ mixtures. f) the peak area ratios of C−O to C=O. The increment indicates the formation of hydrogen bonds.


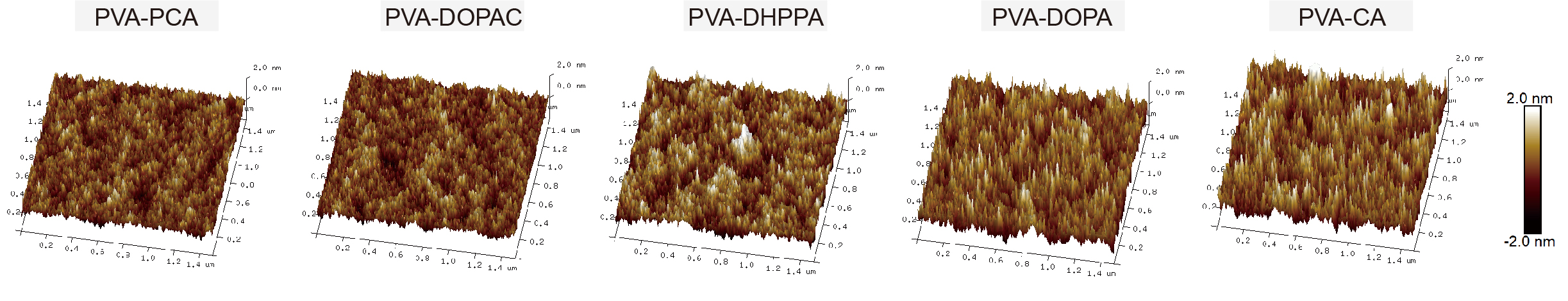


**Figure S13.** Three-dimensional topographic AFM images of the surfaces of five adhesive systems. (Scaning area size =1.6×1.6µm).


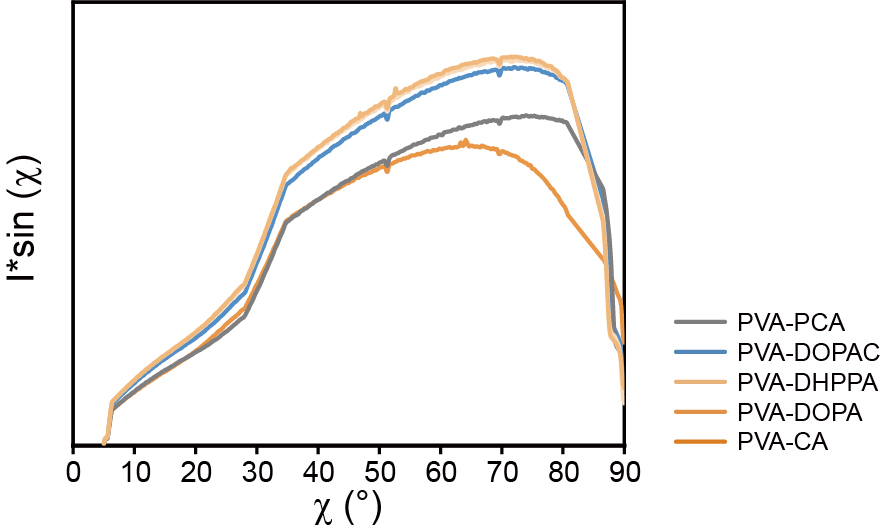


**Figure S14.** Pole figures of five catechol hydrogels films obtained from the integrated intensity of their 2D-GIWAXS images multiplied by a factor of sin (χ) for Ewald geometrical correction.


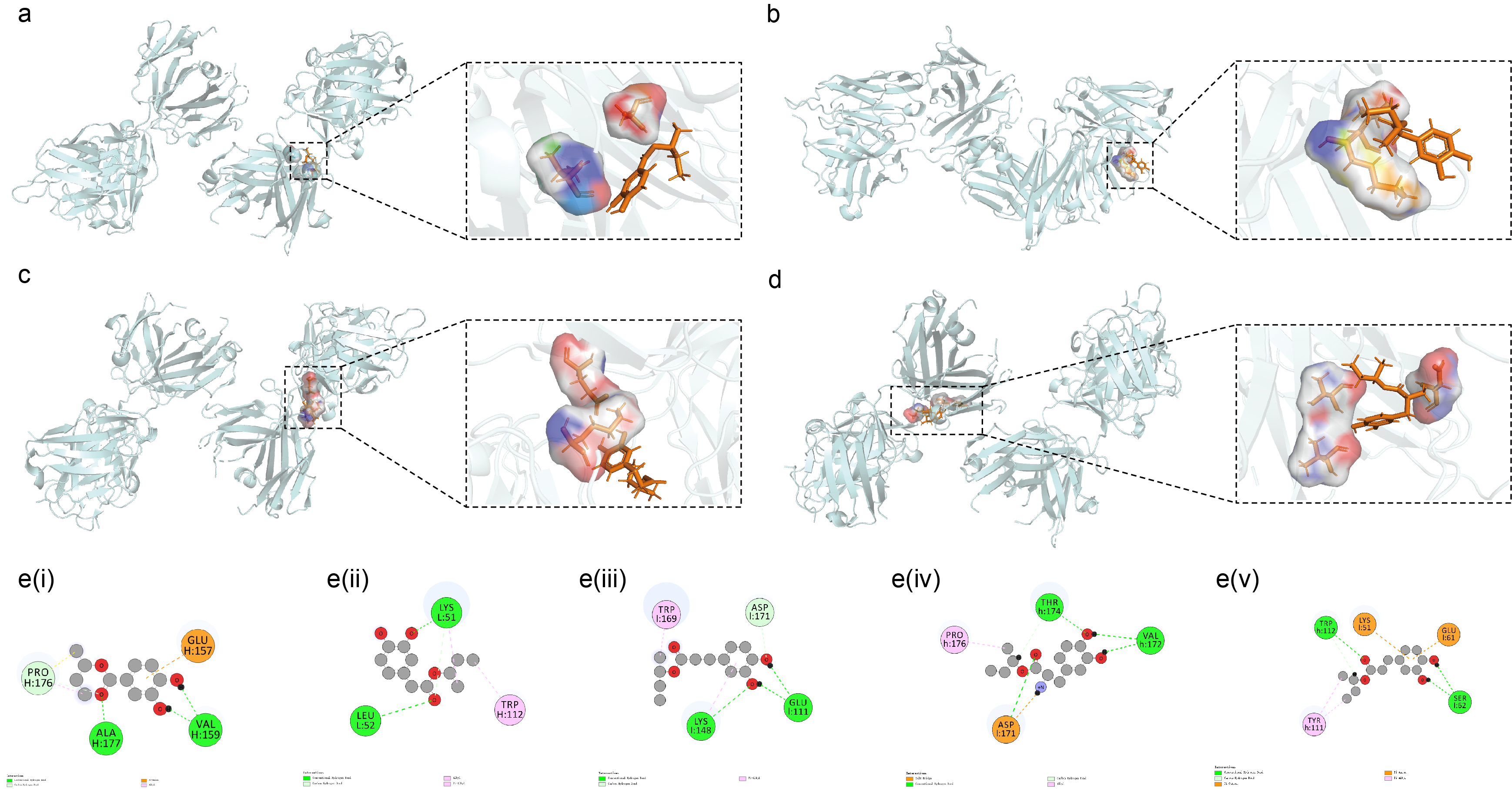


**Figure S15.** 3D schematic diagram of the interaction between PVA-PCA a), PVA-DOPAC b), PVA-DHPPA c), PVA-DOPA d) and MUC1. e) The 2D interaction diagrams of the interaction between five catechol hydrogels and MUC1.

**
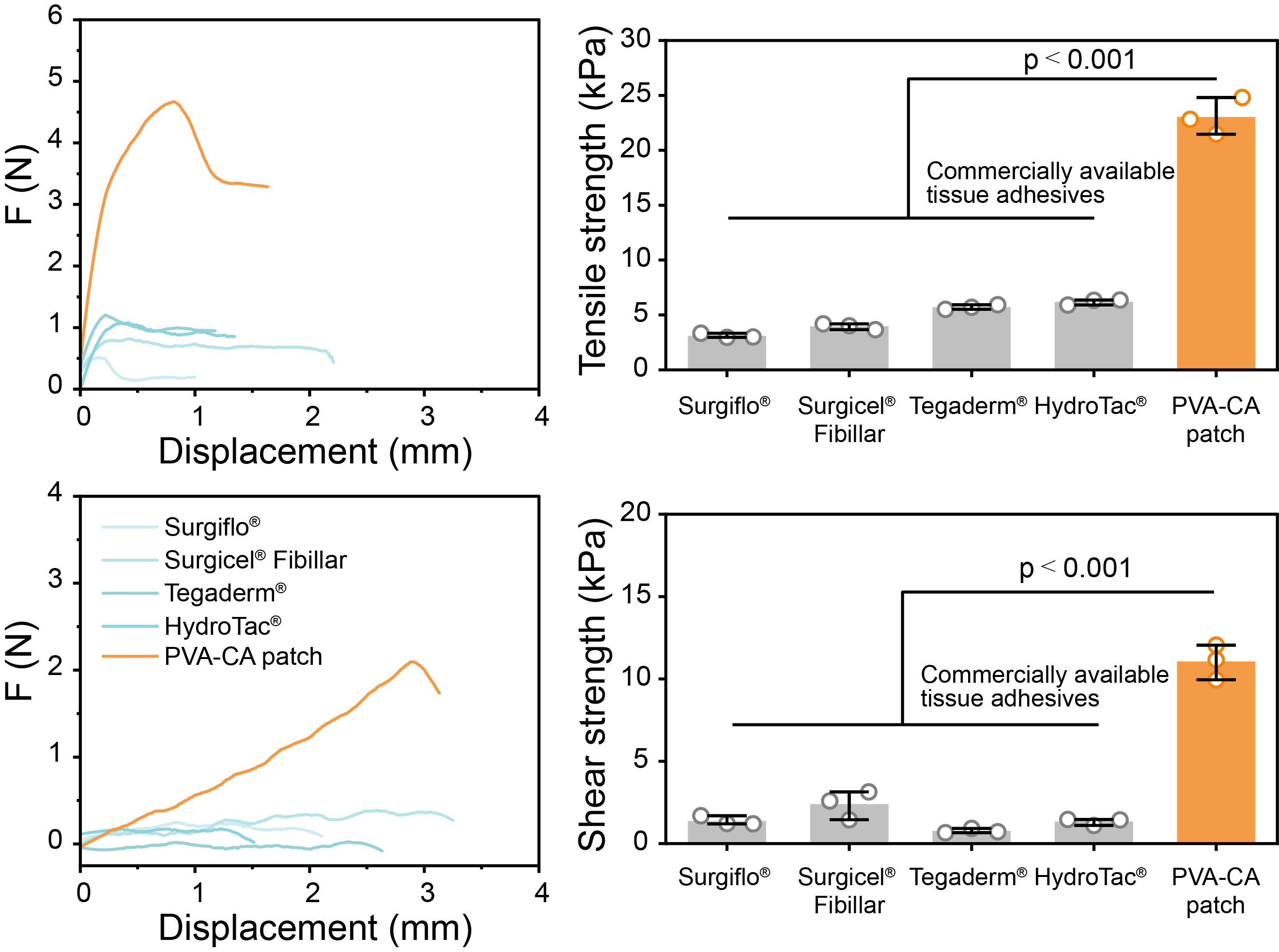
**

**Figure S16.** a) Force-displacement curves of the tensile test. b) Tensile strength of commercially available tissue adhesives and PVA-CA patch on two side of procine skins (n = 3 independent samples). c) Force-displacement curves of the lap shear test. d) Shear strength of commercially available tissue adhesives and PVA-CA patch on one side of procine skins (n = 3 independent samples). Error bars, mean ± SD. P values are determined via one-way ANOVA followed by Tukey’s post-hoc test for c and d.


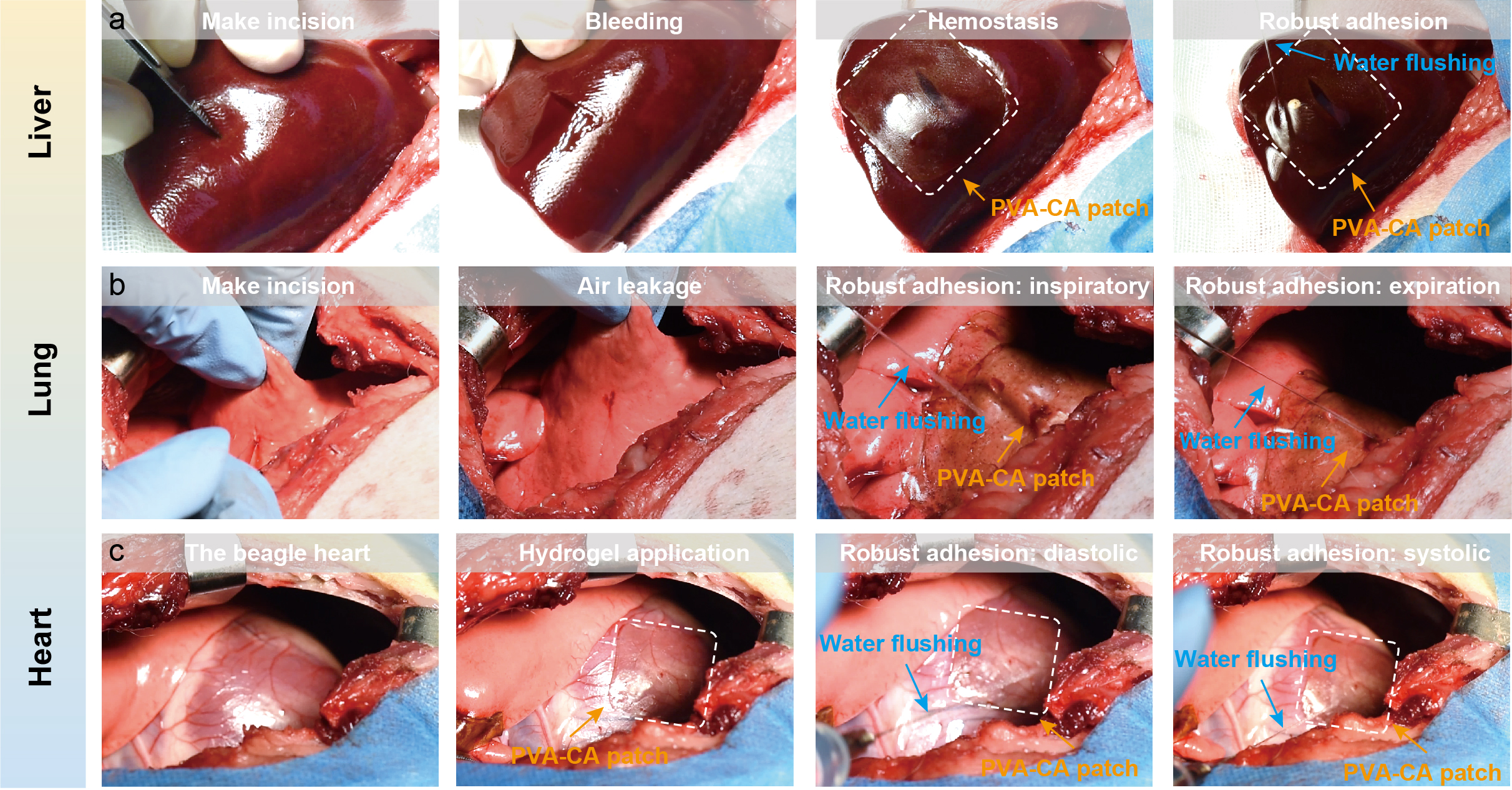


**Figure S17.** In vivo haemostatic sealing in Beagle dogs injury bleeding model. a) Photographs of the hemostasis in liver incision model of Beagle dogs using PVA-CA patch. b) Photographs of the hemostasis in pulmonary incision model of Beagle dogs using PVA-CA patch. c) The PVA-CA patch can be firmly attached to the surface of the beating heart.

**
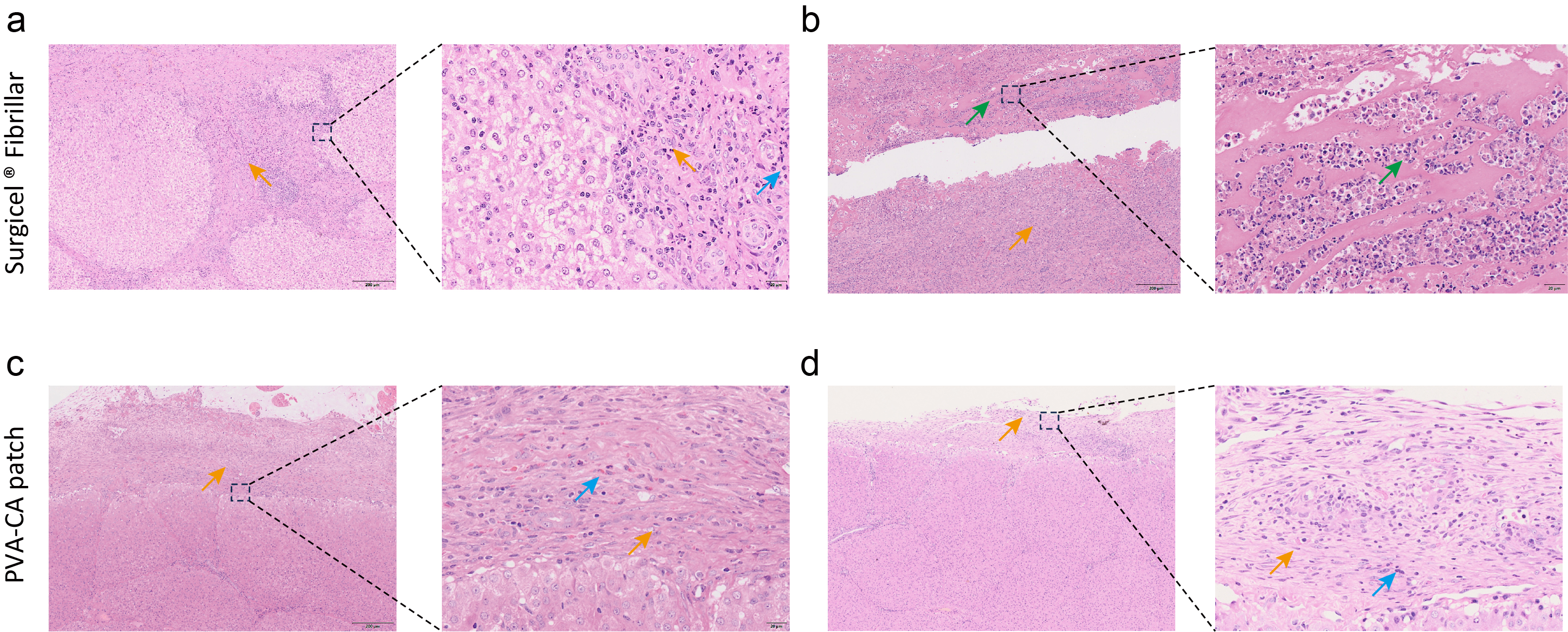
**

**Figure S18.** H&E staining of liver 1 weeks after haemostatic treatment. Yellow arrows indicate fibrous hyperplasia, blue arrows indicate inflammatory cell infiltration, and green arrows indicate necrotic liver cells.


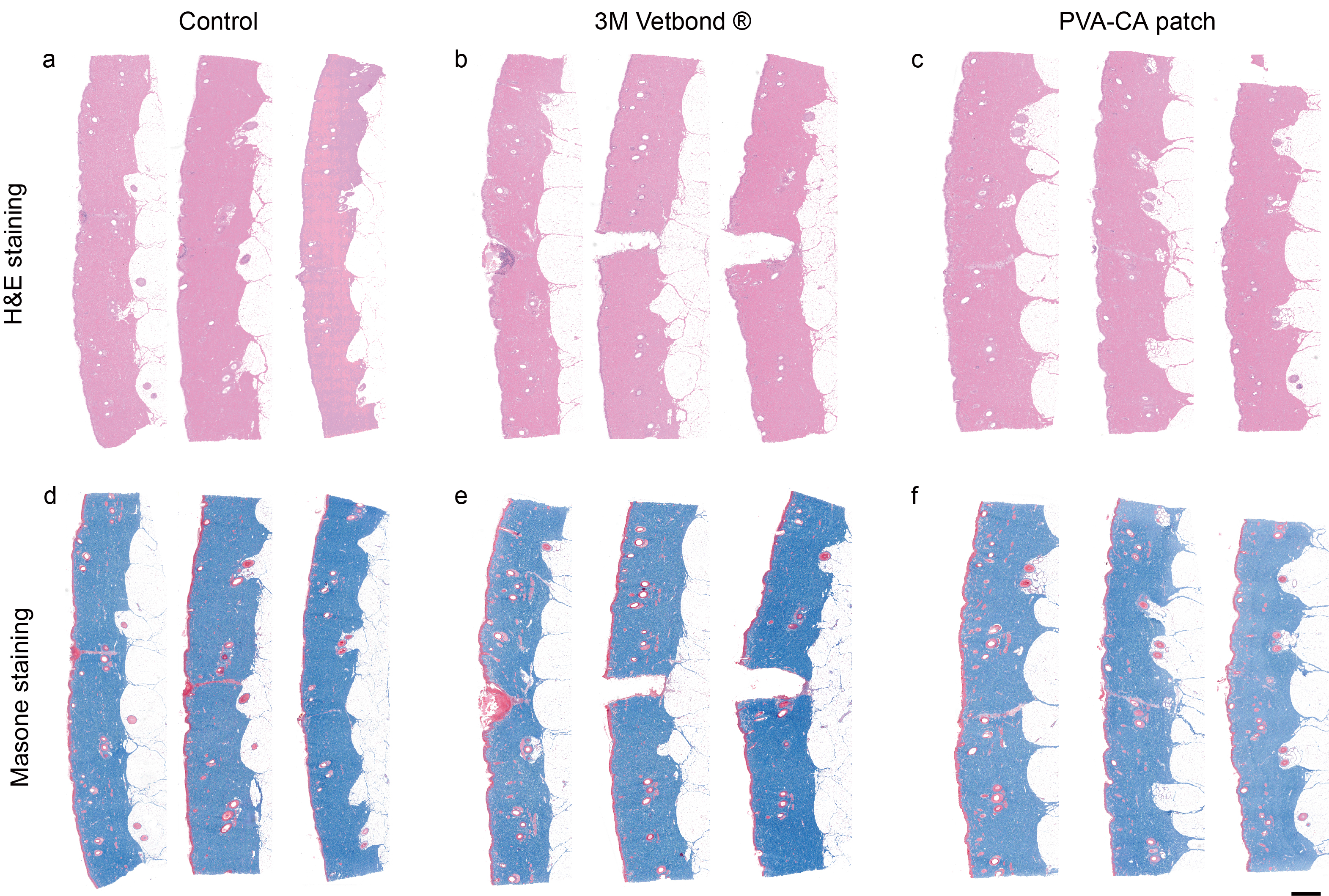


**Figure S19.** H&E staining and Masone staining of skin wounds 1 week later. Scale bars: 1 mm.


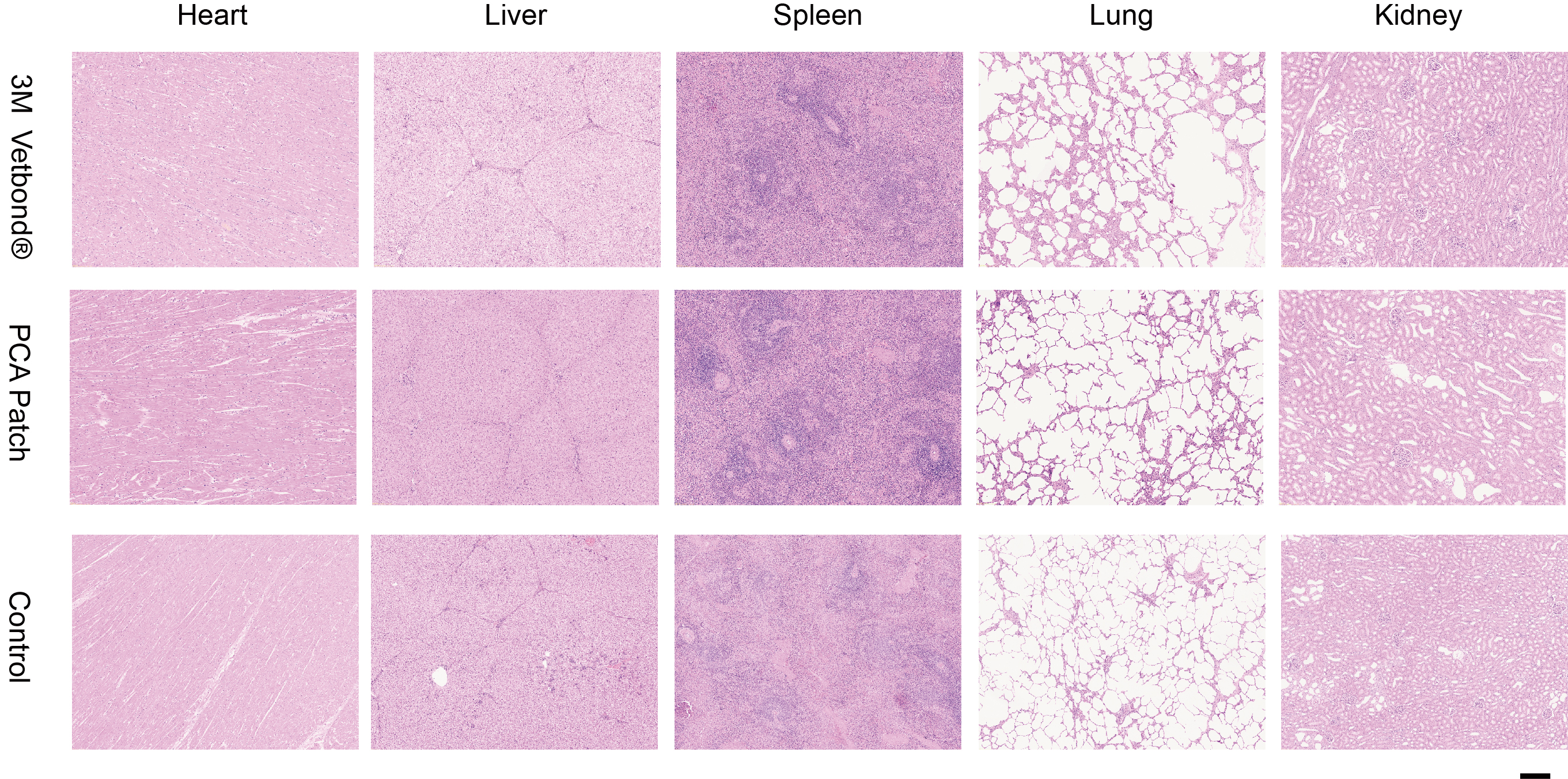


**Figure S20.** H&E staining of major organs (heart, liver, spleen, lung, and kidney) after different treatment in the backs of Bama minipigs for 7 days. Scale bars: 200 μm.

1. **Supplementary Tables**

**Table S1.** Degree of catechol substitution in the adhesive systems conjugates and the grafting efficiency calculated from the ¹H NMR results. Data are presented as means ± standard deviations (SDs) (n = 3 independent samples per group; one-way ANOVA,Tukey’s post-hoc test).

| **Groups** | **Samples** | **Esterification degree of PVA calculated from ^1^H-NMR (%)** | **Grafting Efficiency (%)** |
| --- | --- | --- | --- |
| 1 | PVA-PCA | 9.0±0.6 | 36.1±2.6 |
| 2 | PVA-DOPAC | 8.8±0.3 | 35.2±1.2 |
| 3 | PVA-DHPPA | 9.2±0.5 | 36.9±1.8 |
| 4 | PVA-DOPA | 9.3±0.7 | 37.2±2.6 |
| 5 | PVA-CA | 9.2±0.5^a^ | 37.1±2.0^a^ |

^a^ ns, no significant difference compared with Groups 1-4.

**Table S2.** Statistical parameters describing AFM modulus distribution: mean, SD, raw-data CV, and Gaussian-fit CV.

| **Samples** | **Mean ± SD (MPa)** **^a^** | **CV_raw_ (%)^b^** | **Xc ± σ**  **(GPa)** | **CV_fit_ (%) ^c^** |
| --- | --- | --- | --- | --- |
| PVA-PCA | 2511.65±225.13 | 8.96 | 2.490±0.001 | 0.040 |
| PVA-DOPAC | 4073.31±390.41 | 9.58 | 4.074±0.002 | 0.049 |
| PVA-DHPPA | 4543.13±525.17 | 11.56 | 4.24±0.004 | 0.094 |
| PVA-DOPA | 4616.4±499.46 | 10.82 | 4.443±0.004 | 0.090 |
| PVA-CA | 5636.55±616.04 | 10.93 | 5.568±0.003 | 0.054 |

^a^ Mean and SD were calculated from all AFM modulus pixels.
^b^ CV_raw_ represents the coefficient of variation calculated directly from pixel statistics (SD/mean).
^c^ CV_fit​_ represents the Gaussian-fit coefficient of variation derived from σ/xc.

1. **Legends for movies S1 to S9**

Movie S1. Sealing of a water-leaking stomach

Movie S2. Sealing of a fluid-leaking porcine artery

Movie S3. Sealing of a fluid-leaking porcine small intestine

Movie S4. Hemostasis in pulmonary incision model of Bama minipig using PVA-CA patch

Movie S5. Hemostasis in liver incision model of Bama minipig using PVA-CA patch

Movie S6. Hemostasis in liver incision model of Bama minipig using Surgicel® Fibrillar

Movie S7. Hemostasis in pulmonary incision model of Beagle dog using PVA-CA patch

Movie S8. Hemostasis in liver incision model of Beagle dog using PVA-CA patch

Movie S9. The PVA-CA patch can be firmly attached to the surface of the beating heart

1. **Supplementary References**

[1] P. J. Stephens, F. J. Devlin, C. F. Chabalowski, M. J. Frisch, The Journal of Physical Chemistry 1994, 98, 11623.

[2] a) S. Grimme, J. Antony, S. Ehrlich, H. Krieg, The Journal of Chemical Physics 2010, 132, 154104; b) S. Grimme, S. Ehrlich, L. Goerigk, Journal of Computational Chemistry 2011, 32, 1456.

[3] a) F. Weigend, R. Ahlrichs, Physical Chemistry Chemical Physics 2005, 7, 3297; b) J. Tomasi, B. Mennucci, R. Cammi, Chemical Reviews 2005, 105, 2999.

[4] W. Humphrey, K. Dalke A Fau - Schulten, K. Schulten, Journal of Molecular Graphics 1996; 14(1):33-8, 27-8. doi: 10.1016/0263-7855(96)00018-5.

[5] T. Lu, F. Chen, Journal of Computational Chemistry 2012 15;33(5):580-92. doi: 10.1002/jcc.22885.
